# Forensic Interview Techniques in Child Sexual Abuse Cases: A Scoping Review

**DOI:** 10.1177/15248380231177317

**Published:** 2023-06-05

**Authors:** Delfina Fernandes, João P. Gomes, Pedro B. Albuquerque, Marlene Matos

**Affiliations:** 1 Psychology Research Centre (CIPsi), School of Psychology, University of Minho, Portugal

**Keywords:** child sexual abuse, forensic interview, scoping review, testimony, victims

## Abstract

Child sexual abuse (CSA) is widely recognized as a global public health problem with negative consequences for victims, their families, and society. The child’s testimony is essential to the case outcome, given the frequent absence of physical or biological evidence of the abusive acts. Thus, the child forensic interview plays a decisive role in criminal investigation. The present scoping review aims to identify and describe the judicial procedures for collecting CSA victims’ testimony using an evidence-based approach and a structured methodology. The review followed Preferred Reporting Items of Systematic Reviews and Meta-Analysis-Scoping Review guidelines. Studies were identified through manual reference checking and in four electronic databases: PsycARTICLES, PubMed, SCOPUS, and Web of Science. In all, 146 studies were identified according to the defined inclusion criteria, that is, empirical studies identifying judicial procedures to collect CSA victims’ testimony, published in English or Portuguese. In total, 30 different forensic interview procedures to collect the child victim’s testimony were found. The National Institute for Child Health and Human Development investigative interview protocol was the most frequently mentioned. Despite the variety of protocols, it was possible to conclude that they have a similar general structure. This review also identified gaps in interviewing practices with CSA victims. The scoping review corroborates the importance of forensic interviews with CSA victims, stating its implications for criminal investigation, the legal system, and the child’s recovery process.

Child sexual abuse (CSA)^1^ is a global public health problem that devastates victims, their families, and society (Goodyear-Brown et al., 2012; Hailes et al., 2019). CSA involves various types of criminal behavior operationalized in different crime typologies depending on the legal system of each country. A common definition characterizes CSA as the involvement of any person under the age of 18 years in sexual activity for which he or she is not developmentally prepared and is unable to give consent. The abuser can be any person in a position of trust or power over the child victim. Furthermore, CSA ranges from acts with contact between the abuser and the victim (i.e., with or without sexual intercourse) to acts without contact between them (e.g., exhibitionist acts; World Health Organization, 2017; WHO, 2022a). Therefore, CSA can be conceptualized as a traumatic experience not only because of the behaviors and dynamics it involves, but also because it occurs at a developmental level of particular vulnerability, which can negatively affect several domains of the child’s functioning (Kimberg & Wheeler, 2019).

According to global victimization prevalence data between 1980 and 2008, the number of CSA victims was about 180 girls and 76 boys out of 1000 (Stoltenborgh et al., 2011). Currently, data from the WHO show that one out of five women and one out of 13 men report having been sexually abused under the age of 17 years (WHO, 2022b). These data suggest that the prevalence of CSA victimization has increased. This growth is due to people’s increased awareness of this problem and the greater ease with which children have access to Information and Communication Technologies, most likely enhancing online sexual victimization, which can later expand to the offline world. Thus, the increasing prevalence of CSA victimization causes deep social concern and, consequently, more scientific research on this topic is needed.

In CSA cases, children’s testimony is vital because of the frequent absence of physical or biological evidence of the abusive acts, making them the only witnesses of the crime (Lamb et al., 2018). Such evidence is rare, given the usual lengthy temporal gap between the victimization experience and its disclosure. In addition, children’s reluctance to disclose sexual abuse results from different factors, namely, their relationship with the abuser, abuse severity, their age at the onset of abuse and at the time of the interview, and the quality of support provided by the caregiver (Lamb et al., 2018; Lippert et al., 2009; Wallis & Woodworth, 2020, 2021). Since the testimony of CSA victims is fundamental to the case outcome, the forensic interview plays a decisive role in the criminal investigation.

A forensic interview aims to elicit details about the victimization experience that are unique to the child, which can be important in the decision-making of different judicial entities (Bracewell, 2018; Steele, 2012). The information gathered from the forensic interviews usually refers to a particular type of memory—episodic memory—which refers to the memory for information that occurred in a specific time and space (Tulving, 2002). Several aspects should be addressed in the forensic interview to maximize the quality of the child’s information and mitigate the victims’ adverse consequences. First, the forensic interview needs to be sensitive to the child’s developmental characteristics and idiosyncrasies, such as the child’s age at the moment of the interview, language proficiency, intellectual ability, information-processing skills, and the existence of a developmental disorder (Lamb et al., 2011; Lamb et al., 2018). Second, there are factors associated with the forensic interview that should also be considered, such as the delay between the victimization experience and the interview, the number of interviews that the child has done, and the type of questions asked, where open-ended and non-suggestive questions should be used (Lamb et al., 2011; Lamb et al., 2018). Finally, the interviewer’s characteristics involved when conducting forensic interviews. For this purpose, interviewers must have adequate technical-scientific knowledge, training, and supervision (e.g., in evidence-based best interviewing practices and trauma-informed care; Classen & Clark, 2017; Lamb et al., 2011; Lamb et al., 2018), and ensure a supportive and responsive attitude for children, attending to their developmental level (Lamb et al., 2011; Lamb et al., 2018; Saywitz et al., 2011).

Forensic interviews that do not consider the abovementioned characteristics and are characterized by inappropriate practices can lead to negative consequences for the criminal investigation and the victims, namely, reluctance to disclose the abusive experience (Ettinger, 2022), reduced credibility of the child’s testimony (Johnson & Shelley, 2014; Lamb et al., 2018), increased risk of harm (Lamb et al., 2011), and risk of secondary victimization (i.e., suffering inflicted on the victim by the justice system’s response; Classen & Clark, 2017; Substance Abuse and Mental Health Services Administration [SAMHSA], 2014).

Although previous research has highlighted the importance of forensic interviewing in collecting testimony from CSA victims, the weaknesses of this population, and the best practices that interviewers should follow to overcome these challenges, identified real-world judicial procedures for collecting CSA victims’ testimony through an evidence-based approach and a structured methodology remains unknown. For this purpose, we conducted a scoping review, representing the first direct demonstration to fill this research gap.

A scoping review is useful for mapping the available evidence on a specific topic and answering much broader questions than a systematic review, usually undertaken to answer a specific question (Munn et al., 2018). Therefore, this scoping review examines the following research question: Which judicial procedures are used to collect the testimony of CSA victims? Specifically, this study aims to (1) identify and summarize the judicial procedures to collect the testimony of CSA victims; (2) reflect on the evolution of procedures used; and (3) recognize and reflect on the gaps still existing in the daily practices of real-world forensic interviews.

## Method

### Protocol and Registration

The scoping review protocol follows the Preferred Reporting Items of Systematic Reviews and Meta-Analysis (PRISMA) methodology and its extensions for Scoping Reviews (PRISMA-ScR; Tricco et al., 2018). This scoping review was registered on OSF REGISTRIES (reference: osf.io/e29x8).

### Eligibility Criteria

Studies were considered for inclusion if they (a) report findings from an empirical study (i.e., not literature reviews, theoretical articles, and commentaries or letters to editors); (b) were written in English or Portuguese; and (c) identify the type of approach used in judicial procedures to collect the testimony of CSA victims. Gray literature was also considered for inclusion, namely, unpublished master’s or doctoral theses, or chapters of books presenting empirical studies. No restrictions regarding the year of publication, publication status, research design, or methodology were used.

For a study to be excluded, one or more of the following criteria need to be presented: (a) studies addressing clinical interviews to assess the impact of alleged abusive experiences; (b) studies addressing tools to assess the credibility of the child’s testimony; (c) studies addressing the collection of child testimony in trials since in these cases the questions of attorneys and prosecutors are intended to assess mainly the credibility of the child testimony focusing on option-posing and suggestive questions.

### Information Sources and Search Process

MeSH and other terms combined with Boolean Operators (OR and AND) were used to create the following search equation: (interview* OR “forensic interview*” OR “interview guidelines” OR “judicial proce*” OR “criminal proce*” OR “investigati* proce*” OR “investigative interview*” OR “court proce*” OR statement* OR protocol* OR inquir* OR testify* OR testimon*) AND (child* OR adolescen* OR juvenile* OR youth* OR teen* OR young OR minor* OR toddler* OR infan*) AND (“sexual abuse” victim* OR “sexual assault” victim* OR porn* victim* OR molest* victim* OR “internet sex* offend” victim* OR “sexual act*” victim* OR “sexually offensive conduct” victim* OR “sexual exploitation” victim* OR incest victim* OR rape victim* OR “sexual maltreatment” victim* OR sexting victim*). In April 2022, the equation was run by two reviewers with an MSc in Applied Psychology into four electronic databases searching by title, abstract, and keywords: PsycARTICLES, PubMed, SCOPUS, and Web of Science. In addition, we checked the reference lists of several studies and specialized interdisciplinary journals.

### Study Selection

Identified studies were imported into Rayyan software (Ouzzani et al., 2016), and the duplicates were deleted. Afterwards, the two reviewers independently read the titles and abstracts, and the papers were selected for full-text analysis. All possible reviewer’s disagreements were discussed between them until a consensus was reached.

### Data Charting Process

The two reviewers independently charted data from the included studies using a standardized data extraction sheet in Excel to collect the following topics: (a) study identification (i.e., title, author(s), year of publication, and journal); (b) victim sample characteristics (i.e., sample size, gender, age, ethnicity, and disabilities); (c) victimization characterization (i.e., type of victimization, reported incident(s), victim–abuser relationship, setting of occurrence and frequency of violence); (d) interviewer characteristics (i.e., sample size, gender, age, ethnicity, profession, training, and number of interviewers per interview); (e) forensic interview procedures (i.e., setting of the interview, type of interview, the delay between the incidents and interview, number of sessions, duration, and record); (f) measures; (g) type of outcome; and (h) main findings.

After the data charting process, the frequency count and respective percentage of each forensic interview procedure identified were calculated. Subsequently, the description of each type of interview was noted and extracted. One reviewer extracted these topics, and the other reviewer verified the process and information. All reviewer’s disagreements were solved after discussion and consensus.

### Synthesis of Results

Included studies were reviewed in a qualitative synthesis presented in the section “Results.” Findings were presented in a narrative and table format. However, the description of each forensic interview procedure was shown only in a table format, and the number of sessions or interviews was described only in a narrative format. Key findings were summarized and highlighted their significance.

## Results

### Selection of Evidence Sources

**Figure 1.**
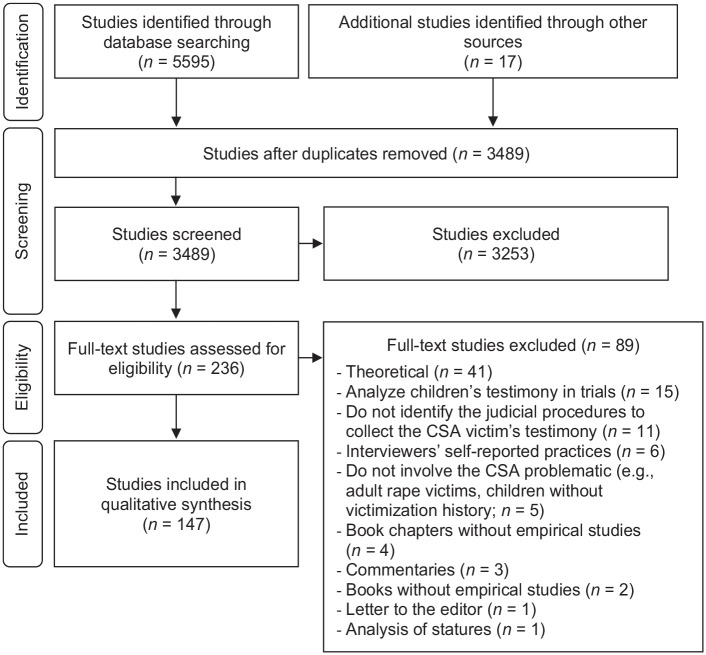
PRISMA flow diagram of search strategy for scoping review. PRISMA = Preferred Reporting Items of Systematic Reviews and Meta-Analysis.

A total of 5,595 studies were identified from the database search, and 17 other studies were located from manual reference checking. After removing duplicates, 3,489 were screened based on title and abstract. From this analysis, 3,253 did not meet the eligibility criteria and were therefore excluded. The full text of 236 studies was assessed for eligibility according to inclusion and exclusion criteria. In the final process, 147 studies were included in this scoping review, and data were extracted from each. Figure 1 presents the PRISMA flow diagram illustrating the number of included studies in each of the selection processes and the rationale for the studies’ inclusion or exclusion. References of identified studies are included in Supplemental Appendix A.

### Characteristics of Evidence Sources

The characteristics of included studies (i.e., author(s), year of publication, type of victimization, sample characteristics, interviewer profession, and forensic interview description) are illustrated in Supplemental Appendix B.

#### Study identification

The publication year range of included studies varied between 1986 and 2022. The studies were conducted in 18 different countries, with the most significant part of them being conducted in Israel (*n* = 43).

#### Victims sample characteristics

The sample size across all the included studies ranged from one (Orbach & Lamb, 1999, 2001) to 40430 (Hershkowitz, Lamb, & Horowitz, 2007). Five studies address the interviewers’ training to collect the CSA victims’ testimony, and therefore a sample size of victims was not applied (Cheung, 1997; Cheung & Boutté-Queen, 2010; Lafontaine & Cyr, 2016a; Smith et al., 2009; Yi et al., 2017).

The victims’ age in the included studies varied between 2 and 50 years. Five studies only reported the average age that ranged between 5.83 and 11.94 years (Duron, 2018a, 2018b; Korkman et al., 2006; Lippert et al., 2009; Santtila et al., 2004) and eight studies report neither the range nor the average age of the sample (Bracewell, 2018; Cheung, 1997; Cheung & Boutté-Queen, 2010; Hershkowitz, Fisher, et al., 2007; Lafontaine & Cyr, 2016a; Smith et al., 2009; Westcott et al., 2006; Yi et al., 2017).

Eleven selected studies analyzed sexual victimization in children and adults with a mental health diagnosis: intellectual disabilities (ID), autism spectrum disorder (ASD), attention deficit hyperactivity disorder (ADHD), developmental disabilities, anxiety, depression, cognitive impairment and/or an unspecific disability (Åker & Johnson, 2020; Anderson et al., 2014; Cantlon et al., 1996; Cederborg & Lamb, 2008; Cederborg et al., 2008; Cederborg et al., 2011; Hershkowitz, Lamb, Horowitz, 2007; Hlavka, 2014; Lee & Kim, 2020; Melkman et al., 2017; Westcott et al., 2006). Two of the included studies analyzed the forensic interview in a specific ethnic group (Aboriginal children; Hamilton et al., 2016a, 2016b).

#### Victimization characterization

Of the 147 included studies, 106 addressed only CSA victimization. In these cases, the reported incidents involved sexual exposure (exhibitionism/voyeurism), fondling of sexual organs over the clothes, fondling of sexual organs under the clothes, and oral, anal, and/or vaginal penetration. In addition, 34 addressed CSA, physical abuse, and/or other types of victimization as neglect. Physical abuse was characterized by physical harm from being hit with and/or without an object. Of the remaining studies, two addressed sexual assault (e.g., rape, indecent image crimes), two human trafficking for sexual exploitations, one internet-related CSA, and in two cases the predominant type of victimization was CSA. However, no information on all incidents was reported (Feltis et al., 2010; Henderson & Lyon, 2020).

#### Interviewer profession

From all the studies, the interviews were collected by police officers (*n* = 72), youth investigators^2^ (*n* = 42), social workers (*n* = 18), psychologists (*n* = 11), forensic interviewers (*n* = 10), child protective service workers (*n* = 5), Child Advocacy Centers professionals (*n* = 4), psychiatrists (*n* = 3), clinical mental healthcare professionals (*n* = 2), judges (*n* = 2), criminologist (*n* = 1), registered nurses with experience in pediatrics or mental health (*n* = 1), or a sheriff (*n* = 1). In three studies, the interviewers’ profession was not reported. In one of the included studies, the authors specified that the interview was conducted jointly by a police officer and a social worker.

### Forensic Interview Procedures

The delay between the abusive incident (or last abusive incident in multiple incident cases) as reported by the child and the first interview was indicated in 31 studies and ranged from 0 days (Cederborg et al., 2000; Hershkowitz et al., 1998; Hershkowitz et al., 2001; Hershkowitz et al., 2002; Orbach et al., 2000) to 10 years (Leander, 2010). However, one included study investigated the amount of time between the first alleged abusive experiences and the first time an adult found out about the abuse, which ranged from 1 month up to several years (Malloy et al., 2011). Most studies defined this delay as the time necessary for the child to disclose the alleged abusive experiences to someone.

#### Frequency of forensic interview procedures

In the review, 30 different forensic interview procedures with CSA victims were identified. The main aim of some included studies was to test the effectiveness of an interview protocol training and therefore compare interviews conducted before and after training on that protocol. In these cases, we consider the pre-training interviews as forensic interview procedures in which no protocols or guidelines were followed. The frequency count and percentage of the forensic interview procedures are presented in Table 1.

**Table 1.** Frequency Count and Percent Frequency of the Interview Types.

Interview Types	Frequency Count	% Frequency
NICHD investigative interview protocol	69	37.30
No specific guidelines or protocols were followed	40	21.62
MoGP	12	6.49
Revised NICHD investigative interview protocol	9	4.86
No specific protocols were followed—stepwise procedures through progressive broad stages	8	4.32
SIM framework	6	3.24
ABE investigative interview protocol	3	1.62
CornerHouse RATAC semi-structured forensic interview protocol	3	1.62
CSAIP	3	1.62
SACD	3	1.62
NICHD investigative interview protocol and HFD with structured questions	2	1.08
NICHD investigative interview protocol with MCR techniques	2	1.08
NICHD investigative interview protocol with the drawing of the alleged abusive experiences	2	1.08
APSAC protocol	2	1.08
SI model	2	1.08
CACs of Texas protocol	2	1.08
Structured interview protocol with an interview at the office and a follow-up interview at the scene of the alleged crime	2	1.08
A scripted protocol for rapport building: Direct	2	1.08
A scripted protocol for rapport building: Open ended	2	1.08
NICHD investigative interview protocol with modifications adapting to the developmental and emotional needs of the child	1	0.54
NICHD investigative interview protocol with PCR techniques	1	0.54
CornerHouse RATAC semi-structured forensic interview protocol with narrative event practice rapport techniques	1	0.54
CornerHouse RATAC semi-structured forensic interview protocol with SACD	1	0.54
CSAIP with SACD and/or HFD	1	0.54
10-Step protocol	1	0.54
SCWI model	1	0.54
Guidance on joint investigative interviewing of child witnesses	1	0.54
Child interview protocol guide of the Children’s House	1	0.54
Allegation blind interview technique with progression from open-ended to leading questions	1	0.54
Allegation-informed interview technique with progression from open-ended to leading questions	1	0.54
Total	185	100.00

*Note.* ABE = Achieving Best Evidence; APSAC = American Professional Society on the Abuse of Children; CACs = Children’s Advocacy Centers; CSAIP = Child Sexual Abuse Interview Protocol; HFD = Human Figure Diagram; MCR = Mental Context Reinstatement; MoGP = Memorandum of Good Practice; NICHD = National Institute for Child Health and Human Development; PCR = Physical Context Reinstatement; RATAC = Rapport, Anatomy Identification, Touch inquiry, Abuse scenario, and Closure; SACD = Sexually Anatomical Correct Dolls; SCWI = Specialist Child Witness Interviewing; SI = Sequential Interview; SIM = Standard Interview Method.

National Institute for Child Health and Human Development (NICHD) investigative interview protocol was the most frequent interview for collecting the CSA victims’ testimony (frequency count = 69; 37.30%). The other methods of collecting the CSA victims’ testimony were the absence of specific guidelines or protocols to conduct forensic interviews (frequency count = 40; 21.62%), Memorandum of Good Practice (MoGP; frequency count = 12; 6.49%), revised NICHD investigative interview protocol (frequency count = 9; 4.86%), absence of specific protocols to conduct the interview; however, stepwise procedures were followed through progressive broad stages (frequency count = 8; 4.32%), Standard Interview Method framework (frequency count = 6; 3.24%), Achieving Best Evidence investigative interview protocol (frequency count = 3; 1.62%), CornerHouse Rapport, Anatomy identification, Touch inquiry, Abuse scenario, and Closure (RATAC) semi-structured forensic interview protocol (frequency count = 3; 1.62%), Child Sexual Abuse Interview Protocol (CSAIP; frequency count = 3; 1.62%), Sexually Anatomical Correct Dolls (SACD; frequency count = 3; 1.62%), NICHD investigative interview protocol using also the Human Figure Diagrams (HFD) with structured questions (frequency count = 2; 1.08%), NICHD investigative interview protocol with Mental Context Reinstatement techniques (frequency count = 2; 1.08%), NICHD investigative interview protocol with the drawing of the alleged abusive experiences (frequency count = 2; 1.08%), American Professional Society on the Abuse of Children protocol (frequency count = 2; 1.08%), Sequential Interview model (frequency count = 2; 1.08%), CACs of Texas protocol (frequency count = 2; 1.08%), structured interview protocol with an interview at the office and a follow-up interview at the scene of the alleged crime (frequency count = 2; 1.08%), a scripted protocol to rapport building: Direct (frequency count = 2; 1.08%), a scripted protocol to rapport building: Open ended (frequency count = 2; 1.08%), NICHD investigative interview protocol with modifications adapting to the developmental and emotional needs of the child (frequency count = 1; 0.54%), NICHD investigative interview protocol with Physical Context Reinstatement techniques (frequency count = 1; 0.54%), CornerHouse RATAC semi-structured forensic interview protocol with narrative event practice rapport techniques (frequency count = 1; 0.54%), CornerHouse RATAC semi-structured forensic interview protocol with SACD (frequency count = 1; 0.54%), CSAIP with SACD (frequency count = 1; 0.54%), 10-Step protocol (frequency count = 1; 0.54%), Specialist Child Witness Interviewing model (frequency count = 1; 0.54%), guidance on joint investigative interviewing of child witnesses (frequency count = 1; 0.54%), child interview protocol guide of the Children’s House (frequency count = 1; 0.54%), allegation blind interview technique with progression from open-ended to leading questions (frequency count = 1; 0.54%), and allegation informed interview technique with progression from open-ended to leading questions (frequency count = 1; 0.54%).

#### Description of forensic interview procedures

The description of each forensic interview procedure is shown in Table 2.

**Table 2.** Description of the Interview Types.

Interview Types	Description
NICHD investigative interview protocol	This protocol is structured in 10 phases: (1) introduction (e.g., interviewer presentation, interview purpose explanation); (2) ground rules (e.g., truth and lies; transfer of control); (3) rapport building; (4) episodic memory training (i.e., narrative event practice); (5) transition to substantive phase; (6) investigative incidents through free recall; option to take a break; (7) focused questions about information not already mentioned followed by open-ended prompts; (8) disclosure information; (9) closure; and (10) neutral topic.
Revised NICHD investigative interview protocol	The revised version covers the same phases of the standard version (i.e., NICHD protocol); however, it was designed to increase children’s emotional comfort and reduce children’s reluctance during investigative interviews. For this, in the revised version, the rapport building is done before explaining ground rules and the episodic memory training and encourages continuous rapport building and supportive interviewing, even if necessary multiple sessions.
NICHD investigative interview protocol and HFD with structured questions	In this case, the NICHD protocol is followed by structured questions in which reference is made to an unclothed (frontal and dorsal), gender-neutral outline drawing. The questioning starts with a directive recall prompt that is followed by alternating yes–no questions. Open-ended free-recall prompts were used to elicit further information that had not yet been mentioned.
NICHD investigative interview protocol with modifications adapting to the developmental and emotional needs of the child	NICHD protocol with the following modifications: (1) pre-substantive and substantive phases spread across more than one interview for reluctant children, with psychological distress, or reporting multiple incidents requiring more time to recall greater amounts of information; (2) free drawing for reluctant or distressed children (no symbolic interpretation); (3) in the absence of disclosure, the touch survey should be used.
NICHD investigative interview protocol with MCR techniques	The NICHD protocol includes MCR techniques in the pre-substantive and substantive phases. The first MCR technique is provided in episodic memory training. In the substantive phase, the same instruction is given before the child is asked to “tell everything” about the abusive experience. When the child reported multiple incidents, the MCR instruction is repeated for each one.
NICHD investigative interview protocol with PCR techniques	The pre-substantive phase of NICHD protocol is completed in the interviewer’s office, but soon as the children made an allegation, they are invited to accompany the interviewer to the scene of the alleged crime, where they are interviewed about substantive issues.
NICHD investigative interview protocol with the drawing of the alleged abusive experiences	In this case, the NICHD protocol is followed, however, after the interviewer had probed the children’s memory of the alleged event using open-ended questions, he gave the children a blank sheet of paper, a pencil, and a rubber eraser and ask the child to draw what happened to her during 7 to 10 minutes. Then the interviewer moved from open-ended to focused questions. The drawings were not interpreted.
MoGP	The MoGP is a general comprehensive guidance (without specific examples) that describes in detail what should be done before, during, and after investigative interviews of children. The MoGP indicates that forensic interviews should include five phases: (1) rapport building (i.e., explaining the ground rules; truth, and lies); (2) free narrative; (3) open-ended questions; (4) closed questions; and (5) closure.
ABE investigative interview protocol	The ABE protocol is a successor of MoGP and is guidance with recommendations to identify the needs of vulnerable witnesses or victims, to plan, and prepare for the interview, to conduct the interview, and to prepare victims for the court process. The interview is typically structured in two major phases: (1) rapport building and (2) free recall followed by open-ended questioning.
CornerHouse RATAC semi-structured forensic interview protocol	This protocol follows three guiding principles: interviews should be person-centered; interviews should be semi-structured; and interviewers should use open-ended prompts, avoid leading and suggestive questioning, and must be unbiased. The protocol is structured in four phases: (1) rapport building, (2) seek information, (3) explore statements, and (4) end respectfully.
CornerHouse RATAC semi-structured forensic interview protocol with narrative event practice rapport techniques	This protocol is different from the traditional version of CornerHouse RATAC protocol in that in this case the children are questioned more thoroughly about a specific event to narrative event practice (i.e., episodic memory training), with more open-ended prompts.
CornerHouse RATAC semi-structured forensic interview protocol with SACD	The CornerHouse RATAC protocol is used in the traditional manner and following a child’s verbal disclosure, an interviewer could choose to introduce anatomical dolls to the child to show and descript their victimization experiences.
APSAC protocol	This protocol is structured in seven phases: (1) introduction (e.g., interviewer presentation, interview purpose explanation); (2) ground rules (e.g., truth, lies, and oath; transfer of control); (3) episodic memory training (i.e., narrative event practice); (4) introduction of the topic of concern; (5) substantive questions; (6) if necessary, physical evidence can be presented to the child; and (7) closure.
SI model	SI splits the interview into more than one session, follows a funnel approach, and uses multiple interviews. This interview is structured in four phases: (1) introduction (e.g., interviewer presentation; explanation of ground rules); (2) rapport building; (3) free narrative account with open-ended questions at the start and directive questions when the first narrative is completed; and (4) closure.
CACs of Texas protocol	This protocol is structured in six phases: (1) preparation (e.g., interviewer presentation, showing the child the interviewer room); (2) rapport building; (3) ground rules (e.g., truth, lies, and oath); (4) introduction of the topic of concern; (5) detail gathering (i.e., fact-finding through free recall and the exploration of alternative hypotheses); and (6) closure.
SIM framework	The SIM framework closely resembles the NICHD protocol. The interview is divided into six phases: (1) introduction; (2) ground rules; (3) rapport building; (4) introduction of the topic of concern; (5) free narrative account; and (6) questioning exhausting the information provided.
10-Step protocol	The 10-Step protocol is a revision of NICHD protocol and is outlined in four phases: (1) instructions (i.e., «do not know» instruction; «do not understand» instruction; «you are wrong» instruction; ignorant interviewer; truth instruction); (2) rapport building with narrative practice; (3) allegation; and (4) closing.
SACD	In interviews with SACD, the interviewer ensured the general rules for an investigative interview with children in a stepwise manner. The SACD is introduced in the substantive phase in which the interviewer prompted children to disclosures of abuse. The dolls are used as demonstration aids or in a playful manner to elicit details of the alleged abuse
SCWI model	The SCWI model follows the PEACE framework (i.e., Planning and Preparation, Engage and Explain, Account, Closure, and Evaluation of the Interview) and is closely modeled on NICHD protocol. The SCWI is structured in three phases: (1) engage and explain (e.g., introduction; explanation of ground rules; narrative event practice); (2) account (starting with open-ended prompts); and (3) closure.
Structured interview protocol with an interview at the office and a follow-up interview at the scene of the alleged crime	The interview starts in the office with an introduction (e.g., the importance of telling the truth), then the phases of rapport building and episodic memory training are followed. After this, the interviewer introduces the substantive issues, and following the children had made an allegation, they were asked to accompany the interviewer to the scene of the alleged crime. There the interviewer asks the children to tell everything they remember about the alleged crime. The interviewer starts with open-ended invitations, then open-ended prompts, cue questions and focused, non-suggestive questions were asked only if some crucial information is missing. In the end, interview closure is assured.
Guidance on joint investigative interviewing of child witnesses	This guidance was developed by Scottish Executive, and it is structured in five phases: (1) introduction; (2) rapport building; (3) practice narrative interview; (4) free recall and questioning with funnel-shaped hierarchical structure; and (5) closure.
Child interview protocol guide of the Children’s House	This guide is broadly based on the NICHD protocol, and it is applied in Iceland. The interview phases in the Children’s House guide are the follows: (1) introduction (e.g., interviewer presentation; interview purpose explanation); (2) ground rules (e.g., do not understand instruction); (3) child’s development and understanding of basic concepts (e.g., days, months, body parts names); (4) truth/lies; (5) practice interview; (6) introduction the topic under investigation; (7) free narrative; (8) questioning and clarification; and (9) closure.
A scripted protocol for rapport building: Direct	This interview starts with an introduction by the interviewer. In the rapport building phase that followed, interviewers followed a direct script. In this script, the interviewer uses focused prompts to elicit information about the child’s family, school, and celebration of a recent holiday (e.g., “Did you turn your books in on the last day of school?”). Then the interviewer focuses on substantive issues under investigation, starting with an invitation to elicit a free recall from the child, then moving on to non-suggestive questioning.
A scripted protocol to rapport building: Open ended	This interview starts with an introduction by the interviewer. In the rapport building phase that followed, interviewers followed an open-ended script. In this script, the interviewer uses open-ended prompts to elicit information about the child’s family, school, and celebration of a recent holiday (e.g., “tell me everything you did on the last day of school”). Then the interviewer focuses on substantive issues under investigation, starting with an invitation to elicit a free recall from the child, then moving on to non-suggestive questioning.
Allegation blind interview technique with progression from open-ended to leading questions	In this interview technique, before interviewing, the interviewer had knowledge only of the child’s names for their body parts and the names of family members. The allegation blind concept parallels asking interview questions on a progression from open-ended to leading questions. This technique is favorable for cases in which the files contain erroneous information.
Allegation-informed interview technique with progression from open-ended to leading questions	In this interview technique, interviewers knew allegation information from having interviewed siblings, comments made by parents, agencies, or other sources. The allegation-informed concept parallels asking interview questions on a progression from open-ended to leading questions.
No specific guidelines or protocols were followed	In these interview types, no specific guidelines or protocols were followed.
No specific protocols were followed—Stepwise procedures through progressive broad stages	In these interview types, no specific guidelines or protocols were followed; however, the interviewers were knowledgeable about the recommendations on how to conduct investigative interviews with children and used stepwise procedures through progressive broad stages, such as introduction, rapport building, free narrative, questioning, and closure.

*Note.* ABE = Achieving Best Evidence; APSAC = American Professional Society on the Abuse of Children; CACs = Children’s Advocacy Centers; HFD = Human Figure Diagram; MCR = Mental Context Reinstatement; MoGP = Memorandum of Good Practice; NICHD = National Institute for Child Health and Human Development; PCR = Physical Context Reinstatement; RATAC = Rapport, Anatomy Identification, Touch inquiry, Abuse scenario, and Closure; SACD = Sexually Anatomical Correct Dolls; SCWI = Specialist Child Witness Interviewing; SI = Sequential Interview; SIM = Standard Interview Method.

The number of sessions or interviews was also analyzed in this review. Of the included studies, 126 analyzed a single interview per child. Regarding the remaining studies, six analyzed single interviews with multiple sessions with breaks between them, that is, multiphase interviews (Baugerud et al., 2020; Hershkowitz & Terner, 2007; Hershkowitz et al., 1998; Katz & Hershkowitz, 2010, 2013; Orbach et al., 2000). In Hershkowitz et al.’s (1998) and Orbach et al.’s (2000) studies, there is no information about the break time between the two sessions. In the study of Baugerud et al. (2020), 52.2% of the children had one session, 45.4% had two sessions, 1.4% had three sessions, and 1% had four sessions with two to three breaks between the sessions. In this case, the first break lasted between 45 and 60 minutes and the final break lasted from 5 to 10 minutes for legal representatives to have the chance to suggest any remaining questions. In Hershkowitz and Terner’s (2007) study, the interviewer paused the interview for 30 minutes, and in the remaining studies, the break lasted between 7 and 10 minutes (Katz & Hershkowitz, 2010, 2013).

In the remaining 15 included studies, some children had more than one interview to collect the testimony about their victimization experiences. The number of interviews ranged from one to two (*M* = 1.1, *SD* = 0.3; Sumampouw et al., 2019), one to three (*M* = 2.0, *SD* = 0.9; Leander, 2010), one to four (Åker & Johnson, 2020), one to six (*M* = 2.3, *SD* = 1.6; Korkman et al., 2006), one to six (*M* = 3.4, *SD* = 1.5; Lamb & Fauchier, 2001), one to six (*M* = 2.3, *SD* = 1.6; Santtila et al., 2004), one to seven (*M* = 3.2, *SD* = 1.4; Azzopardi et al., 2014), two to five (*M* = 2.52; Waterhouse et al., 2016), more than one (Alves et al., 2019; Cederborg et al., 2008), two for all children in the study (Blasbalg et al., 2021; Hershkowitz et al., 2021; Katz, 2014; Waterhouse et al., 2018), and three for all children in the study (Patterson & Pipe, 2009). In two of the studies, the repeated interviews with the child were conducted by the same interviewer as the previous one (Azzopardi et al., 2014; Waterhouse et al., 2018). In Waterhouse et al.’s (2016) study, 60% of the repeated interviews were also conducted by the same interviewer. The interval between interviews across all these studies ranged from 1 day (Azzopardi et al., 2014; Blasbalg et al., 2021; Cederborg et al., 2008; Waterhouse et al., 2016) to 368 days (Waterhouse et al., 2016).

## Discussion

The main objective of this scoping review was to identify and summarize real-world judicial procedures for collecting the testimony of CSA victims using an evidence-based approach and a structured methodology.

The review identified 30 different forensic interview procedures with CSA victims. However, it should be noted that some protocols were used in a large proportion of studies developed by the same authors, with different outcomes analyzed. As a result, those protocols present a higher frequency, but it is not so frequent in terms of their use by different authors.

Although the review indicates a broad spectrum of guidelines or protocols for collecting the CSA victims’ testimony, we conclude that their general structure is similar. Specifically, forensic interviews have a semi-structured format, that is, each interview is adapted to the developmental needs of the child. Furthermore, forensic interviews are generally structured in six phases: (1) introduction, where, for example, the interviewer introduces himself and other persons who may be in the room, explain the interview purpose, and explain the ground rules (i.e., truth and lies; transfer of control); (2) rapport building, in which the interviewer elicits information about the child’s family, school, and personal preferences; (3) episodic memory training, where the purpose is for the interviewer to assess the child’s development and understanding of basic concepts and to prepare the child to narrate an event; (4) free recall, only if victimization was first verbalized by the child, where the interviewer starts with an open-ended invitation demanding the child to recall information about the alleged abusive experience(s); (5) questioning, in which the interviewer progresses to focused questions to clarify or elicit more information about the alleged abusive experience(s); and (6) closure, where, for example, the child has the opportunity to ask questions to the interviewer and the interview is closed on a neutral topic. These stages can be modified or eliminated according to the child’s developmental needs and can be complemented by strategies such as HFD, SACD, touch survey, and drawings.

Although most of the studies used a standardized approach, this review concluded that there are still professionals in certain countries who use non-evidence-based practices. This is one of the most serious problems in forensic interviewing, with implications for the quantity and quality of the information provided by the child (Alonzo-Proulx & Cyr, 2016; Cyr & Lamb, 2009; Lamb et al., 2018; Yi et al., 2016), assessment of child’s credibility (Hershkowitz, Fisher, et al., 2007; Johnson & Shelley, 2014), case outcomes (Pipe et al., 2013), and the possibility of revictimization (SAMHSA, 2014).

The included studies also used a different number of sessions or interviews to collect the CSA victims’ testimony. Regarding the number of sessions, studies analyzed the collection of testimony in a single interview with several sessions on the same day. The purposes of multiphase interviews were to (a) enable the child to relax and feel safe; (b) assess the immediate re-interviewing on the quantity or quality of details provided by the child; (c) determine whether environmental contextual cues provided by visits to the scenes of alleged abusive experiences would facilitate the recall of information by CSA victims; or (d) analyze the effect of drawing on the amount of information reported later by the child and feelings of self-efficacy. In the latter, the interviewer does not interpret what is elaborated in the pauses.

Concerning the repetition of interviews on different days, this happens for three main reasons: (a) when the child did not disclose the alleged abusive experiences on the first interview; (b) when the child did not provide critical abuse-related information during the previous session(s); or (c) to analyze how the child recounted the alleged facts. Overall, the included studies addressing re-interviewing concluded that an additional interview seems to be effective in disclosing new information relevant to the criminal investigation, mainly about other events than those mentioned or denied in the previous session(s) (Azzopardi et al., 2014; Hershkowitz & Terner, 2007; Lamb & Fauchier, 2001; Waterhouse et al., 2016). Furthermore, these studies reported the utility of the multiple interviews model for children due to developmental (e.g., ID) or motivational limitations (e.g., reluctant children), requiring more time to build rapport with the interviewer and more than one opportunity to report their experiences (Cederborg et al., 2008; Hershkowitz et al., 2021; Katz, 2014; Leander, 2010; Patterson & Pipe, 2009). Thus, the studies highlighted the importance of adjusting the number of forensic interviews or sessions and the retention interval between them based on the child’s individual needs and the contextual circumstances of the abusive events, maintaining the best practices recommendations of question format in all interviews (i.e., free memory recall and open-ended questions), avoiding duplicative interviews (La Rooy et al., 2010).

In addition, the included studies mentioned that the initial training and the use of guidelines and protocols are insufficient to increase the quality of information collected from the child. Ongoing training and supervision of interviewers are critical aspects of a successful forensic interview. This training should include components such as developmental characteristics of children (e.g., memory, suggestibility), conceptual and empirical support for all the phases of an interview, and behaviors throughout the interview (Lamb et al., 2002). Moreover, role-playing and individual, detailed, written feedback are also important aspects (Cyr et al., 2012; Lamb et al., 2002; Price & Roberts, 2011).

Furthermore, the training of interviewer professionals in trauma-informed care approach is essential since they can have a significant impact on the recovery and mental well-being of CSA victims. Thus, it is expected that the justice system professionals reveal knowledge, attitudes, and practices based on trauma-informed care to avoid retraumatizing CSA victims namely, how to deal with children’s victimization issues in respectful and equitable ways, understand the impact of trauma on different domains of functioning, and how this affects the way that children engage with the justice system (Classen & Clark, 2017; SAMHSA, 2022). Finally, it was possible to conclude that the same procedures used to collect the testimony of CSA victims can be generalized to vulnerable groups such as preschool children, children with a mental health diagnosis (e.g., ID, ASD, ADHD, anxiety, depression), and to adults with IDs. Similarly, two studies examined a procedure for collecting CSA victims’ testimony on a specific ethnic group, discussing the implications for this group. In addition, forensic interview procedures developed for collecting CSA victims’ testimony have become widespread, showing that they can be applied to other types of child maltreatment and CSA witnesses. These results indicated that investigating CSA victims has revealed cultural sensitivity and attention to intersectionality.

Despite the promising results of this review, it is appropriate to recognize some potential limitations. First, data collection only included studies written in English or Portuguese, so we cannot be sure if all the studies on this topic have been included. However, as a broad spectrum of forensic interview procedures was obtained in this review, we consider that we identified all the possibilities to collect the CSA victims’ testimony. In future research, it would be helpful to extend the current findings by examining each procedure’s effectiveness, empirical validity, and reliability in collecting the CSA victims’ testimony.

### Conclusion and Implications

The scientific literature has shown that children have developmental difficulties that may hinder their ability to describe abusive experiences. It is also known that these limitations can be overcome by best practices followed by interviewers, for example, the use of interview techniques and protocols. This is not only important for the legal process and criminal investigation, but also a significant step for the child’s recovery process (Katz et al., 2014).

However, the identification of real-world judicial procedures for collecting CSA victims’ testimony through an evidence-based approach and a structured methodology remains unknown. For this purpose, we conducted the scoping review, representing the first direct demonstration to fill this research gap. Thus, the review allows a comprehensive and descriptive approach to CSA victims’ testimony collection procedures.

The review identified a broad spectrum of forensic interview procedures to collect the CSA victims’ testimony. However, we conclude that their basic structure is similar. Specifically, the included studies highlight the importance of starting with an introduction where the interviewer provides some explanations. Then, it is necessary to rapport building with the child and practice the child’s narrative of events. Subsequently, the interviewer should allow the child to freely recall the alleged abusive experiences progressing to questioning through a funnel approach. Finally, the interview should be ended neutrally. These stages should be adapted according to the child’s individual needs. The review also revealed that forensic interviews identified are culturally sensitive and attend to intersectionality. Thus, the results of this review can be generalized to different samples.

Finally, although some countries adhere to best practices evidenced by the scientific literature, this review also identified gaps in interviewing practices with CSA victims. The lack of adherence in some settings to standardized and evidence-based guidelines or protocols, the lack of sensitivity to children’s capacity level, and the lack of interviewer ongoing training and supervision are leading to inadequate forensic interview practices. As we have already mentioned, this has consequences for the child and the criminal investigation, but it also has additional economic costs to the justice system. These constraints could be offset by institutional efforts to prevent this serious problem, such as investment in training and ongoing supervision of evidence-based best practices among professionals who collect CSA victims’ testimony in criminal investigative procedures (e.g., police officers, psychologists, judges, prosecutors; Tables 3 and 4).

**Table 3.** Critical Findings.

• The review identified 30 different forensic interview procedures with CSA victims. • NICHD investigative interview protocol was the most frequent interview for collecting the CSA victims’ testimony. • Forensic interviews are generally structured in six phases: (1) introduction; (2) rapport building; (3) episodic memory training; (4) free recall; (5) questioning; and (6) closure. These stages can be modified or eliminated according to the child’s developmental needs and can be complemented by strategies such as HFD, SACD, touch survey, and drawings. • Forensic interviews identified revealed cultural sensitivity and attended to intersectionality. Thus, the research data can be generalized to different samples. • Gaps in interviewing practices with CSA victims were identified.

*Note*. CSA = child sexual abuse; HFD = Human Figure Diagram; NICHD = National Institute for Child Health and Human Development; SACD = Sexually Anatomical Correct Dolls.

**Table 4.** Summary of Implications for Practice, Policy, and Research.

• Professionals conducting forensic interviews should use evidence-based practices to collect the CSA victims’ testimony. • Inadequate forensic interviewing practices have consequences for the child and the criminal investigation. This could be mitigated by training and ongoing supervision among professionals who collect CSA victims’ testimony in criminal investigative procedures and trauma-informed care approach. • The constraints arising from inadequate forensic interviewing practices also have additional economic costs to the justice system. Thus, institutional efforts should be undertaken to prevent these problems. • Research examining each procedure’s effectiveness, empirical validity, and reliability in collecting the CSA victims’ testimony is needed.

*Note*. CSA = child sexual abuse.

## Supplemental Material

sj-docx-1-tva-10.1177_15248380231177317 – Supplemental material for Forensic Interview Techniques in Child Sexual Abuse Cases: A Scoping Review Supplemental material, sj-docx-1-tva-10.1177_15248380231177317 for Forensic Interview Techniques in Child Sexual Abuse Cases: A Scoping Review by Delfina Fernandes, João P. Gomes, Pedro B. Albuquerque and Marlene Matos in Trauma, Violence, & Abuse

sj-docx-2-tva-10.1177_15248380231177317 – Supplemental material for Forensic Interview Techniques in Child Sexual Abuse Cases: A Scoping Review Supplemental material, sj-docx-2-tva-10.1177_15248380231177317 for Forensic Interview Techniques in Child Sexual Abuse Cases: A Scoping Review by Delfina Fernandes, João P. Gomes, Pedro B. Albuquerque and Marlene Matos in Trauma, Violence, & Abuse

## Notes

1. In the present article, the concept of “child,” for the sake of language simplification, will be used in the broad sense, that is, children or adolescents up to 18 years.

2. Youth’s investigators are a specific case from the Israeli Ministry of Welfare and Social Services who have a degree in social work and are the only professionals authorized to conduct forensic interviews with children in Israel.
