# Supplementary material for: Forensic Interview Techniques in Child Sexual Abuse Cases: A Scoping Review
Source: Trauma Violence Abuse. 2023 Jun 5;25(2):1382–96. doi: 10.1177/15248380231177317 (PMC10913353; doi:10.1177/15248380231177317)
Supplement: sj-docx-1-tva-10.1177_15248380231177317 – Supplemental material for Forensic Interview Techniques in Child Sexual Abuse Cases: A Scoping Review [file sj-docx-1-tva-10.1177_15248380231177317.docx]

**Supplemental Appendix A**

**References of identified articles**

Ahern, E. C., Andrews, S. J., Stolzenberg, S. N., & Lyon, T. D. (2015). The productivity of wh-prompts when children testify. *Journal of Interpersonal Violence, 33*(13), 2007-2015. https://doi.org/10.1177/0886260515621084

Ahern, E. C., Hershkowitz, I., Lamb, M. E., Blasbalg, U., & Winstanley, A. (2014). Support and reluctance in the pre-substantive phase of alleged child abuse cictim investigative interviews: Revised versus standard NICHD protocols. *Behavioral Sciences and the Law, 32*(6), 762-774. https://doi.org/10.1002/bsl.2149

Ahern, E. C., & Lamb, M. E. (2017). Children's reports of disclosure recipient reactions in forensic interviews: Comparing the NICHD and MoGP protocols. *Journal of Police and Criminal Psychology, 32*(2), 85-93. https://doi.org/10.1007/s11896-016-9205-x

Ahern, E. C., VanMeter, F., & Lamb, M. E. (2018). A macro-coding perspective: Interviewer support and child comfort in investigative interviews with young alleged victims of sexual abuse. *Children and Youth Services Review, 95,* 361-367. https://doi.org/10.1016/j.childyouth.2018.11.013

Åker, T. H., & Johnson, M. S. (2020). Interviewing alleged victims with mild and moderate intellectual disabilities and autism: A field study of police-investigated cases of physical and sexual abuse in a Norwegian national sample. *Journal of Intellectual Disability Research, 64*(10), 782-792. https://doi.org/10.1111/jir.12771

Aldridge, J., & Cameron, S. (1999). Interviewing child witnesses: Questioning techniques and the role of training. *Applied Developmental Science, 3*(2), 136-147. http://dx.doi.org/10.1207/s1532480xads0302_7

Aldridge, J., Lamb, M. E., Sternberg, K. J., Orbach, Y., Esplin, P. W., & Bowler, L. (2004). Using a human figure drawing to elicit information from alleged victims of child sexual abuse. *Journal of Consulting and Clinical Psychology, 72*(2), 304-316. https://doi.org/10.1037/0022-006X.72.2.304

Alonzo-Proulx, A., & Cyr, M. (2016). Factors predicting central details in alleged child sexual abuse victims' disclosure. *Journal of Forensic Psychology Practice, 16*(3), 129-150. http://doi.org/10.1080/15228932.2016.1172422

Alsaif, D. M., Almadani, O. M., Almoghannam, S. A., Al-Farayedhi, M. A., & Kharoshah, M. A. (2018). Teaching children about self-protection from sexual abuse: Could it be a cause for source monitoring errors and fantasy? (Two case reports). *Egyptian Journal of Forensic Sciences, 8*(27). https://doi.org/10.1186/s41935-018-0058-6

Alves Jr., R. T., Nelson-Gardell, D., Tavares, M., & Young, T. L. (2019). Developing a functional code system to analyze forensic interviews with suspected victims of child sexual abuse. *Child and Adolescent Social Work Journal, 36*(3), 305-316. https://doi.org/10.1007/s10560-019-00612-z

Anderson, G. D., Anderson, J. N., & Gilgun, J. F. (2014). The influence of narrative practice techniques on child behaviors in forensic interviews. *Journal of Child Sexual Abuse, 23*(6), 615-634. https://doi.org/10.1080/10538712.2014.932878

Andrews, S. J., & Lamb, M. E. (2014). The effects of age and delay on responses to repeated questions in forensic interviews with children alleging sexual abuse. *Law and Human Behavior, 38*(2), 171-180. https://doi.org/10.1037/lhb0000064

Azzopardi, C., Madigan, S., & Kirkland-Burke, M. (2014). Sexual abuse forensic evaluation with young children: Program outcomes and predictors of disclosure. *Journal of Child Custody, 11*(4), 304-324. https://doi.org/10.1080/15379418.2014.988901

Baugerud, G.-A., Johnson, M. S., Hansen, H. B., Magnussen, S., & Lamb, M. E. (2020). Forensic interviews with preschool children: An analysis of extended interviews in Norway (2015-2017). *Applied Cognitive Psychology, 34*(3), 654-663. https://doi.org/10.1002/acp.3647

Blasbalg, U., Hershkowitz, I., Lamb, M. E., & Karni-Visel, Y. (2021). Adherence to the revised NICHD protocol recommendations for conducting repeated supportive interviews is associated with the likelihood that children will allege abuse. *Psychology, Public Policy, and Law, 27*(2), 209-220. https://doi.org/10.1037/law0000295

Bracewell, T. E. (2018). Outcry consistency and prosecutorial decisions in child sexual abuse cases. *Journal of Child Sexual Abuse, 27*(4), 424-438. https://doi.org/10.1080/10538712.2018.1474413

Brubacher, S. P., Malloy, L. C., Lamb, M. E., & Roberts, K. P. (2013). How do interviewers and children discuss individual occurrences of alleged repeated abuse in forensic interviews? *Applied Cognitive Psychology, 27*(4), 443-450. https://doi.org/10.1002/acp.2920

Burrows, K. S., Bearman, M., Dion, J., & Powell, M. B. (2017). Children's use of sexual body part terms in witness interviews about sexual abuse. *Child Abuse & Neglect, 65*, 226-235. https://doi.org/10.1016/j.chiabu.2017.02.001

Cantlon, J., Payne, G., & Erbaugh, C. (1996). Outcome-based practice: Disclosure rates of child sexual abuse comparing allegation blind and allegation informed structured interviews. *Child Abuse & Neglect, 20*(11), 1113-1120. https://doi.org/10.1016/0145-2134(96)00100-7

Castelli, P., & Goodman, G. (2014). Children's perceived emotional behavior at disclosure and prosecutors' evaluations. *Child Abuse & Neglect, 38*(9), 1521-1532. https://doi.org/10.1016/j.chiabu.2014.02.010

Cederborg, A.-C., Hultman, E., & La Rooy, D. (2011). The quality of details when children and youths with intellectual disabilities are interviewed about their abuse experiences. *Scandinavian Journal of Psychology, 14*(2), 113-125. https://doi.org/10.1080/15017419.2010.541615

Cederborg, A.-C., La Rooy, D., & Lamb, M. E. (2008). Repeated interviews with children who have intellectual disabilities. *Journal of Applied Research in Intellectual Disabilities, 21*(2), 103-113. https://doi.org/10.1111/j.1468-3148.2007.00372.x

Cederborg, A.-C., & Lamb, M. E. (2008). Interviewing alleged victims with intellectual disabilities. *Journal of Intellectual Disability Research, 52*(1), 49-58. https://doi.org/10.1111/j.1365-2788.2007.00976.x

Cederborg, A.-C., Orbach, Y., Sternberg, K. J., & Lamb, M. E. (2000). Investigative interviews of child witnesses in Sweden. *Child Abuse & Neglect, 24*(10). 1355-1361. https://doi.org/10.1016/S0145-2134(00)00183-6

Cheung, K. M. (1997). Developing the interview protocol for video-recorded child sexual abuse investigations: A training experience with police officers, social workers, and clinical psychologists in Hong Kong. *Child Abuse & Neglect, 21*(3), 273-284. https://doi.org/10.1016/S0145-2134(96)00154-8

Cheung, K. M. (2008). Promoting effective interviewing of sexually abused children: A pilot study. *Research on Social Work Practice, 18*(2), 137-143. https://doi.org/10.1177/1049731507304359

Cheung, M., & Boutté-Queen, N. M. (2010). Assessing the relative importance of the child sexual abuse interview protocol items to assist child victims in abuse disclosure. *Journal of Family Violence, 25*, 11-22. https://doi.org/10.1007/s10896-009-9265-0

Cyr, M., Dion, J., McDuff, P., & Trotier-Sylvan, K. (2012). Transfer of skills in the context of non-suggestive investigative interviews: Impact of structured interview protocol and feedback. *Applied Cognitive Psychology, 26*(4), 516-524. https://doi.org/10.1002/acp.2822

Cyr, M., & Lamb, M. E. (2009). Assessing the effectiveness of the NICHD investigative interview protocol when interviewing French-speaking alleged victims of child sexual abuse in Quebec. *Child Abuse & Neglect, 33*(5), 257-268. https://doi.org/10.1016/j.chiabu.2008.04.002

Davies, G. M., Westcott, H. L., & Horan, N. (2000). The impact of questioning style on the content of investigative interviews with suspected child sexual abuse victims. *Psychology, Crime & Law, 6*(3), 81-97. https://doi.org/10.1080/10683160008410834

Dion, J., & Cyr, M. (2008). The use of the NICHD protocol to enhance the quantity of details obtained from children with low verbal abilities in investigative interviews: A pilot study. *Journal of Child Sexual Abuse, 17*(2), 144-162. https://doi.org/10.1080/10538710801916564

Duron, J. F. (2018a). Legal decision-making in child sexual abuse investigations: A mixed-methods study of factors that influence prosecution. *Child Abuse & Neglect, 79*, 302-314. https://doi.org/10.1016/j.chiabu.2018.02.022

Duron, J. F. (2018b). Searching for truth: The forensic interviewer's use of an assessment approach while conducting child sexual abuse interviews. *Journal of Child Sexual Abuse, 29*(2), 183-204. https://doi.org/10.1080/10538712.2018.1484833

Earhart, B., La Rooy, D. J., Brubacher, S. P., & Lamb, M. E. (2014). An examination of “don’t know” responses in forensic interviews with children. *Behavioral Sciences and the Law, 32*(6), 746-761. https://doi.org/10.1002/bsl.2141

Feltis, B. B., Powell, M. B., Snow, P. C., & Hughes-Scholes, C. H. (2010). An examination of the association between interviewer question type and story-grammar detail in child witness interviews about abuse. *Child Abuse & Neglect, 34*(6), 407-413. https://doi.org/10.1016/j.chiabu.2009.09.019

Gagnon, K., & Cyr, M. (2017). Sexual abuse and preschoolers: Forensic details in regard of question types. *Child Abuse & Neglect, 67*, 109-118. https://doi.org/10.1016/j.chiabu.2017.02.022

Garcia, F. J., Powell, M. B., Brubacher, S. P., Eisenchlas, S. A., & Low-Choy, S. (2022). The influence of transition prompt wording on response informativeness and rapidity of disclosure in child forensic interviews. *Psychology, Public Policy, and Law, 28*(2), 255-266. https://doi.org/10.1037/law0000347

Gudjonsson, G., Sveinsdottir, T., Sigurdsson, J. F., & Jonsdottir, J. (2010). The ability of suspected victims of childhood sexual abuse (CSA) to give evidence. Findings from the Children's House in Iceland. *The Journal of Forensic Psychiatry & Psychology, 21*(4). 569-586. https://doi.org/10.1080/14789940903540784

Hamilton, G., Brubacher, S. P., & Powell, M. B. (2016a). Investigative interviewing of Aboriginal children in cases of suspected sexual abuse. *Journal of Child Sexual Abuse, 25*(4), 363-381. https://doi.org/10.1080/10538712.2016.1158762

Hamilton, G., Brubacher, S. P., & Powell, M. B. (2016b). Expressions of shame in investigative interviews with Australian Aboriginal children. *Child Abuse & Neglect, 51*, 64-71. https://doi.org/10.1016/j.chiabu.2015.11.004

Henderson, H. M., & Lyon, T. D. (2020). Children's signaling of incomprehension: The diagnosticity of practice questions during interview instructions. *Child Maltreatment, 26*(1), 95-104. https://doi.org/10.1177/1077559520971350

Hershkowitz, I. (2001). Children's responses to open-ended utterances in investigative interviews. *Legal and Criminological Psychology, 6*(1), 49-63. https://doi.org/10.1348/135532501168190

Hershkowitz, I. (2002). The role of facilitative prompts in interviews of alleged sex abuse victims. *Legal and Criminological Psychology, 7*(1), 63-71. https://doi.org/10.1348/135532502168388

Hershkowitz, I. (2006). Delayed disclosure of alleged child abuse victims in Israel. *American Journal of Orthopsychiatry, 76*(4), 444-450. https://doi.org/10.1037/0002-9432.76.4.444

Hershkowitz, I. (2009). Socioemotional factors in child sexual abuse investigations. *Child Maltreatment, 14*(2), 172-181. https://doi.org/10.1177/1077559508326224

Hershkowitz, I., Aherm E. C., Lamb, M. E., Blasbalg, U., Karni-Visel, Y., & Breitman, M. (2017). Changes in interviewers' use of supportive techniques during the revised protocol training. *Applied Cognitive Psychology, 31*(3), 340-350. https://doi.org/10.1002/acp.3333

Hershkowitz, I., Fisher, S., Lamb, M. E., & Horowitz, D. (2007). Improving credibility assessment in child sexual abuse allegations: The role of the NICHD investigative interview protocol. *Child Abuse & Neglect, 31*(2), 99-110. https://doi.org/10.1016/j.chiabu.2006.09.005

Hershkowitz, I., Horowitz, D., & Lamb, M. E. (2005). Trends in children's disclosure of abuse in Israel: a national study. *Child Abuse & Neglect, 29*(11), 1203-1214. https://doi.org/10.1016/j.chiabu.2005.04.008

Hershkowitz, I., & Lamb, M. E. (2020). Allegation rates and credibility assessment in forensic interviews of alleged child abuse victims: Comparing the revised and standard NICHD protocols. *Psychology, Public Policy, and Law, 26*(2), 176-184. http://doi.org/10.1037/law0000230

Hershkowitz, I., Lamb, M. E., Blasbalg, U., & Karni-Visel, Y. (2021). The dynamics of two-session interviews with suspected victims of abuse who are reluctant to make allegations. *Development and Psychopathology, 33*(2), 739-747. https://doi.org/10.1017/S0954579420001820

Hershkowitz, I., Lamb, M. E., Horowitz, D. (2007). Victimization of children with disabilities. *American Journal of Orthopsychiatry, 77*(4), 629-635. https://doi.org/10.1037/0002-9432.77.4.629

Hershkowitz, I., Lamb, M. E., & Katz, C. (2014). Allegation Rates in Forensic Child Abuse Investigations: Comparing the Revised and Standard NICHD Protocols. *Psychology, Public Policy, and Law, 20*(3), 336-344. http://doi.org/10.1037/a0037391

Hershkowitz, I., Lamb, M. E., Katz, C., & Malloy, L. C. (2013). Does enhanced rapport-building alter the dynamics of investigative interviews with suspected victims of intra-familial abuse? *Journal of Police and Criminal Psychology, 30*, 6-14. https://doi.org/10.1007/s11896-013-9136-8

Hershkowitz, I., Lamb, M. E., Orbach, Y., Katz, C., & Horowitz, D. (2012). The development of communicative and narrative skills among preschoolers: Lessons from forensic interviews about child abuse. *Child Development, 83*(2), 611-622. https://doi.org/10.1111/j.1467-8624.2011.01704.x

Hershkowitz, I., Lamb, M. E., Sternberg, K. J., & Esplin, P. W. (1997). The relationships among interviewer utterance type, CBCA scores and the richness of children's responses. *Legal and Criminological Psychology, 2*(2), 169-176. https://doi.org/10.1111/j.2044-8333.1997.tb00341.x

Hershkowitz, I., Lanes, O., & Lamb, M. E. (2007). Exploring the disclosure of child sexual abuse with alleged victims and their parents. *Child Abuse & Neglect, 31*(2), 111-123. https://doi.org/10.1016/j.chiabu.2006.09.004

Hershkowitz, I., Orbach, Y., Lamb, M. E., Sternberg, K. J., & Horowitz, D. (2001). The effects of mental context reinstatement on children's accounts of sexual abuse. *Applied Cognitive Psychology, 15*(3), 235-248. https://doi.org/10.1002/acp.699

Hershkowitz, I., Orbach, Y., Lamb, M. E., Sternberg, K. J., & Horowitz, D. (2002). A comparison of mental and physical context reinstatement in forensic interviews with alleged victims of sexual abuse. *Applied Cognitive Psychology, 16*(4), 429-441. https://doi.org/10.1002/acp.804

Hershkowitz, I., Orbach, Y., Lamb, M. E., Sternberg, K. J., & Horowitz, D. (2006). Dynamics of forensic interviews with suspected abuse victims who do not disclose abuse. *Child Abuse & Neglect, 30*(7), 753-769. https://doi.org/10.1016/j.chiabu.2005.10.016

Hershkowitz, I., Orbach, Y., Lamb, M. E., Sternberg, K. J., Horowitz, D., & Hovav, M. (1998). Visiting the scene of the crime: Effects on children's recall of alleged abuse. *Legal and Criminological Psychology, 3*(2), 195-207. https://doi.org/10.1111/j.2044-8333.1998.tb00361.x

Hershkowitz, I., & Terner, A. (2007). The effects of repeated interviewing on children's forensic statements of sexual abuse. *Applied Cognitive Psychology, 21*(9), 1131-1143. https://doi.org/10.1002/acp.1319

Hlavka, H. R. (2014). Legal subjectivity among youth victims of sexual abuse. *Law & Social Inquiry, 39*(1), 31-61. https://doi.org/10.1111/lsi.12032

Hlavka, H. R., Olinger, S. D., & Lashley, J. L. (2010). The use of anatomical dolls as a demonstration aid in child sexual abuse interviews: A study of forensic interviewers' perceptions. *Journal of Child Sexual Abuse, 19*(5), 519-553. https://doi.org/10.1080/10538712.2010.511988

Johnson, M., Magnussen, S., Thoresen, C., Lønnum, K., Burrell, L. V., & Melinder, A. (2015). Best practice recommendations still fail to result in action: A national 10-year follow-up study of investigative interviews in CSA cases. *Applied Cognitive Psychology, 29*(5), 661-668. https://doi.org/10.1002/acp.3147

Karni-Visel, Y., Hershkowitz, I., Lamb, M. E., & Blasbalg, U. (2021). Nonverbal emotions while disclosing child abuse: The role of interviewer support. *Child Maltreatment, 28*(1), 66-75*.* https://doi.org/10.1177/10775595211063497

Kask, K. (2012). Dynamics in using different question types in Estonian police interviews of children. *Applied Cognitive Psychology, 26*(2), 324-329. https://doi.org/10.1002/acp.1831

Katz, C. (2013). Internet-related child sexual abuse: What children tell us in their testimonies. *Children and Youth Services Review, 35*(9), 1536-1542. https://doi.org/10.1016/j.childyouth.2013.06.006

Katz, C. (2014). "Please believe me; I am the biggest liar that exists": Characterising children's recantations during forensic investigations. *Children and Youth Services Review, 43*, 160-166. https://doi.org/10.1016/j.childyouth.2014.05.020

Katz, C. (2015). “Stand by me”: The effect of emotional support on children's testimonies. *British Journal of Social Work, 45*(1), 349-362. https://doi.org/10.1093/bjsw/bct137

Katz, C., & Barnetz, Z. (2014). The behavior patterns of abused children as described in their testimonies. *Child Abuse & Neglect, 38*(6), 1033-1040. https://doi.org/10.1016/j.chiabu.2013.08.006

Katz, C., & Barnetz, Z. (2018). "I know it because it happened to me!" Confrontations of children within forensic investigations. *Child Abuse & Neglect, 82*, 124-133. https://doi.org/10.1016/j.chiabu.2018.06.002

Katz, C., Barnetz, Z., & Hershkowitz, I. (2014). The effect of drawing on children's experiences of investigations following alleged child abuse. *Child Abuse & Neglect, 38*(5), 858-867. https://doi.org/10.1016/j.chiabu.2014.01.003

Katz, C., & Hershkowitz, I. (2010). The effects of drawing on children's accounts of sexual abuse. *Child Maltreatment, 15*(2), 171-179. https://doi.org/10.1177/1077559509351742

Katz, C., & Hershkowitz, I. (2012). The effect of multipart prompts on children's testimonies in sexual abuse investigations. *Child Abuse & Neglect, 36*(11-12), 753-759. https://doi.org/10.1016/j.chiabu.2012.07.002

Katz, C., & Hershkowitz, I. (2013). Repeated interviews with children who are the alleged victims of sexual abuse. *Research on Social Work Practice, 23*(2), 210-218. https://doi.org/10.1177/1049731512467511

Katz, C., Tener, D., & Hindi, I. (2021). "We took turns": How do child victims of intrafamilial child sexual abuse perceive and experience their siblings? *Children and Youth Services Review, 130*. https://doi.org/10.1016/j.childyouth.2021.106227

Katz, C., Tener, D., Marmor, A., Lusky-Weisrose, E., & Mordi, H. (2020). “Yes, my uncle, I’ll do whatever you say”: Experiences of Israeli Muslim Arab children during forensic interviews following child sexual abuse. *Journal of Interpersonal Violence, 37*(5-6), 2465-2489. https://doi.org/10.1177/0886260520943732

Katz, C., Hershkowitz, I., Malloy, L. C., Lamb, M. E., Atabaki, A., & Spindler, S. (2012). Non-verbal behavior of children who disclose or do not disclose child abuse in investigative interviews. *Child Abuse & Neglect, 36*(1), 12-20. https://doi.org/10.1016/j.chiabu.2011.08.006

Katz, C., Paddon, M. J., & Barnetz, Z. (2016). Emotional language used by victims of alleged sexual abuse during forensic investigation. *Journal of Child Sexual Abuse, 25*(3), 243-261. https://doi.org/10.1080/10538712.2016.1137666

Kim, S., Alison, L., & Christiansen, P. (2020). Observing rapport-based interpersonal techniques to gather information from victims. *Psychology, Public Policy, and Law, 26*(2), 166-175. https://doi.org/10.1037/law0000222

Korkman, J., Santtila, P., Drzewiecki, T., & Sandnabba, K. N. (2008). Failing to keep it simple: Language use in child sexual abuse interviews with 3-8-year-old children. *Psychology, Crime & Law, 14*(1), 41-60. https://doi.org/10.1080/10683160701368438

Korkman, J., Santtila, P., & Sandnabba, K. N. (2006). Dynamics of verbal interaction between interviewer and child in interviews with alleged victims of child sexual abuse. *Scandinavian Journal of Psychology, 47*(2), 109-119. https://doi.org/10.1111/j.1467-9450.2006.00498.x

Korkman, J., Santtila, P., Westeraker, M., & Sandnabba, N. K. (2008). Interviewing techniques and follow-up questions in child sexual abuse interviews. *European Journal of Developmental Psychology, 5*(1), 108-128. https://doi.org/10.1080/17405620701210460

Lafontaine, J., & Cyr, M. (2016a). A study of the relationship between investigators’ personal characteristics and adherence to interview best practices in training. *Psychiatry, Psychology and Law, 23*(5), 782-797. https://doi.org/10.1080/13218719.2016.1152925

Lafontaine, J., & Cyr, M. (2016b). The relation between interviewers' personal characteristics and investigative interview performance in a child sexual abuse context. *Police Practice and Research, 18*(2), 106-118. https://doi.org/10.1080/15614263.2016.1242423

Lamb, M. E., & Fauchier, A. (2001). The effects of question type on self-contradictions by children in the course of forensic interviews. *Applied Cognitive Psychology, 15*(5), 483-491. https://doi.org/10.1002/acp.726

Lamb, M. E., & Garretson, E. (2003). The effects of interviewer gender and child gender on the informativeness of alleged child sexual abuse victims in forensic interviews. *Law and Human Behavior, 27*(2), 157-171. https://doi.org/10.1023/A:1022595129689

Lamb, M. E., Hershkowitz, I., Sternberg, K. J., Boat, B., & Everson, M. D. (1996). Investigative interviews of alleged sexual abuse victims with and without anatomical dolls. *Child Abuse & Neglect, 20*(12), 1251-1259. https://doi.org/10.1016/S0145-2134(96)00121-4

Lamb, M. E., Orbach, Y., Hershkowitz, I., Horowitz, D., & Abbott, C. B. (2007). Does the type of prompt affect the accuracy of information provided by alleged victims of abuse in forensic interviews? *Applied Cognitive Psychology, 21*(9), 1117-1130. https://doi.org/10.1002/acp.1318

Lamb, M. E., Orbach, Y., Sternberg, K. J., Aldridge, J., Pearson, S., Stewart, H. L., Esplin, P. W., & Bowler, L. (2009). Use of a structured investigative protocol enhances the quality of investigative interviews with alleged victims of child sexual abuse in Britain. *Applied Cognitive Psychology, 23*(4), 449-467. https://doi.org/10.1002/acp.1489

Lamb, M. E., Sternberg, K. J., & Esplin, P. W. (2000). Effects of age and delay on the amount of information provided by alleged sex abuse victims in investigative interviews. *Child Development, 71*(6), 1586-1596. https://doi.org/10.1111/1467-8624.00250

Lamb, M. E., Sternberg, K. J., Orbach, Y., Esplin, P. W., & Mitchell, S. (2002). Is ongoing feedback necessary to maintain the quality of investigative interviews with allegedly abused children? *Applied Developmental Science, 6*(1), 35-41. https://doi.org/10.1207/S1532480XADS0601_04

Lamb, M. E., Sternberg, K. J., Orbach, Y., Esplin, P. W., Stewart, H., & Mitchell, S. (2003). Age differences in young children’s responses to open-ended invitations in the course of forensic interviews. *Journal of Consulting and Clinical Psychology, 71*(5), 926-934. https://doi.org/10.1037/0022-006X.71.5.926

Lamb, M. E., Sternberg, K. J., Orbach, Y., Hershkowitz, I., & Horowitz, D. (2003). Differences between accounts provided by witnesses and alleged victims of child sexual abuse. *Child Abuse & Neglect, 27*(9), 1019-1031. https://doi.org/10.1016/s0145-2134(03)00167-4

Lamb, M. E., Sternberg, K. J., Orbach, Y., Hershkowitz, I., Horowitz, D., & Esplin, P. W. (2000). The effects of intensive training and ongoing supervision on the quality of investigative interviews with alleged sex abuse victims. *Applied Developmental Science, 6*(3), 114-125. https://doi.org/10.1207/S1532480XADS0603_2

Leach, C., Powell, M. B., Sharman, S. J., & Anglim, J. (2017). The relationship between children's age and disclosures of sexual abuse during forensic interviews. *Child Maltreatment, 22*(1), 79-88. https://doi.org/10.1177/1077559516675723

Leander, L. (2010). Police interviews with child sexual abuse victims: patterns of reporting, avoidance and denial. *Child Abuse & Neglect, 34*(3), 192-205. https://doi.org/10.1016/j.chiabu.2009.09.011

Lee, S., & Kim, J. (2020). Rapport quality in investigative interviews: Effects on open-ended questions and free recall responses. *Police Practice and Research, 22(1), 996-1008.* https://doi.org/10.1080/15614263.2020.1786691

Lewy, J., Cyr, M., & Dion, J. (2015). Impact of interviewers' supportive comments and children's reluctance to cooperate during sexual abuse disclosure. *Child Abuse & Neglect, 43*, 112-122. https://doi.org/10.1016/j.chiabu.2015.03.002

Lindholm, J., Börjesson, M., & Cederborg, A.-C. (2014). “What happened when you came to Sweden?” Attributing responsibility in police interviews with alleged adolescent human trafficking victims. *Narrative Inquiry, 24*(2), 181-199. https://doi.org/10.1075/ni.24.2.01lin

Lindholm, J., Cederborg, A.-C., & Alm, C. (2015). Adolescent girls exploited in the sex trade: Informativeness and evasiveness in investigative interviews. *Police Practice and Research, 16*(3), 197-210. https://doi.org/10.1080/15614263.2014.880839

Lippert, T., Cross, T. P., Jones, L., & Walsh, W. (2009). Telling interviewers about sexual abuse: Predictors of child disclosure at forensic interviews. *Child Maltreatment, 14*(1), 100-113. https://doi.org/10.1177/1077559508318398

Malloy, L. C., Brubacher, S. P., & Lamb, M. E. (2011). Expected consequences of disclosure revealed in investigative interviews with suspected victims of child sexual abuse. *Applied Developmental Science, 15*(1), 8-19. https://doi.org/10.1080/10888691.2011.538616

Malloy, L. C., Brubacher, S. P., & Lamb, M. E. (2013). "Because she's one who listens": Children discuss disclosure recipients in forensic interviews. *Child Maltreatment, 18*(4), 245-251. https://doi.org/10.1177/1077559513497250

Malloy, L. C., Orbach, Y., Lamb, M. E., & Walker, A. G. (2016). "How" and "Why" prompts in forensic investigative interviews with preschool children. *Applied Developmental Science, 21*(1), 58-66. https://doi.org/10.1080/10888691.2016.1158652

Melkman, E. P., Hershkowitz, I., & Zur, R. (2017). Credibility assessment in child sexual abuse investigations: A descriptive analysis. *Child Abuse & Neglect, 67*, 76-85. https://doi.org/10.1016/j.chiabu.2017.01.027

Myklebust, T., & Bjørklund, R. A. (2010). Factors affecting the length of responses in field investigative interviews of children (FIIC) in child sexual abuse cases. *Psychiatry, Psychology and Law, 17*(2), 273-289. https://doi.org/10.1080/13218710903421290

Orbach, Y., Hershkowitz, I., Lamb, M. E., Sternberg, K. J., Esplin, P. W., & Horowitz, D. (2000). Assessing the value of structured protocols for forensic interviews of alleged child abuse victims. *Child Abuse & Neglect, 24*(6), 733-752. https://doi.org/10.1016/s0145-2134(00)00137-x

Orbach, Y., Hershkowitz, I., Lamb, M. E., Sternberg, K. J., & Horowitz, D. (2000). Interviewing at the scene of the crime: Effects on children's recall of alleged abuse. *Legal and Criminological Psychology, 5*(1), 135-147. https://doi.org/10.1348/135532500167930

Orbach, Y., & Lamb, M. E. (1999). Assessing the accuracy of a child's account of sexual abuse: A case study. *Child Abuse & Neglect, 23*(1), 91-98. https://doi.org/10.1016/s0145-2134(98)00114-8

Orbach, Y., & Lamb, M. E. (2000). Enhancing children's narratives in investigative interviews. *Child Abuse & Neglect, 24*(12), 1631-1648. https://doi.org/10.1016/S0145-2134(00)00207-6

Orbach, Y., & Lamb, M. E. (2001). The relationship between within-interview contradictions and eliciting interviewer utterances. *Child Abuse & Neglect, 25*(3), 323-333. https://doi.org/10.1016/S0145-2134(00)00254-4

Orbach, Y., & Lamb, M. E. (2007). Young children's references to temporal attributes of allegedly experienced events in the course of forensic interviews. *Child Development, 78*(4), 1100-1120. https://doi.org/10.1111/j.1467-8624.2007.01055.x

Patterson, T., & Pipe, M.-E. (2009). Exploratory assessments of child abuse: Children's responses to interviewer's questions across multiple interview sessions. *Child Abuse & Neglect, 33*(8), 490-504. https://doi.org/10.1016/j.chiabu.2008.12.012

Peixoto, C. E., Fernandes, R. V., Almeida, T. S., Silva, J. M., La Rooy, D., Ribeiro, C., Magalhães, T., & Lamb, M. E. (2017). Interviews of children in a Portuguese special judicial procedure.  *Behavioral Sciences & the Law, 35*(3), 189-203. https://doi.org/10.1002/bsl.2284

Peixoto, C. E., Ribeiro, C., Fernandes, R. V., & Almeida, T. S. (2016). Forensic interviewing of witnesses in Portugal. In D. Walsh, G. Oxburgh, A. D. Redlich, & T. Myklebust (Eds.), *Contemporary developments and practices in investigative interviewing and interrogation: An international perspective* (pp. 188-198). London: Routledge Press

Phillips, E., Oxburgh, G., Gavin, A., & Myklebust, T. (2012). Investigative interviews with victims of child sexual abuse: The relationship between question type and investigation relevant information. *Journal of Police and Criminal Psychology, 27*, 45-54. https://doi.org/10.1007/s11896-011-9093-z

Pipe, M.-E., Orbach, Y., Lamb, M. E., Abbott, C. B., & Stewart, H. (2013). Do case outcomes change when investigative interviewing practices change? *Psychology, Public Policy, and Law, 19*(2), 179-190. https://doi.org/10.1037/a0030312

Price, E. A., Ahern, E. C., & Lamb, M. E. (2016). Rapport-building in investigative interviews of alleged child sexual abuse victims. *Applied Cognitive Psychology, 30*(5), 743-749. https://doi.org/10.1002/acp.3249

Price, H. L., & Roberts, K. P. (2011). The effects of an intensive training and feedback program on police and social workers' investigative interviews of children. *Canadian Journal of Behavioural Science, 43*(3), 235-244. https://doi.org/10.1037/a0022541

Richardson, E., Stokoe, E., & Antaki C. (2019). Establishing intellectually impaired victims' understanding about 'truth' and 'lies': Police interview guidance and practice in cases of sexual assault. *Applied Linguistics, 40*(5), 773-792. https://doi.org/10.1093/applin/amy023

Santtila, P., Korkman, J., & Sandnabba, K. (2004). Effects of interview phase, repeated interviewing, presence of a support person, and anatomically detailed dolls on child sexual abuse interviews. *Psychology, Crime & Law, 10*(1), 21-35. https://doi.org/10.1080/1068316021000044365

Schaeffer, P., Leventhal, J. M., & Asnes, A. G. (2011). Children's disclosures of sexual abuse: learning from direct inquiry. *Child Abuse & Neglect, 35*(5), 343-352. https://doi.org/10.1016/j.chiabu.2011.01.014

Sim, M. P. Y., & Lamb, M. E. (2013). Children's disclosure of child sexual abuse: How motivational factors affect linguistic categories related to deception detection. *Psychology, Crime & Law, 19*(8), 649-660. http://doi.org/10.1080/1068316X.2012.719621

Smith, R. M., Powell, M. B., & Lum, J. (2009). The relationship between job status, interviewing experience, gender, and police officers' adherence to open-ended questions. *Legal and Criminological Psychology, 14*(1), 51-63. https://doi.org/10.1348/135532507X262360

Sternberg, K. J., Lamb, M. E., Davies, G. M., & Westcott, H. L. (2001). The memorandum of good practice: Theory versus application. *Child Abuse & Neglect, 25*(5), 669-681. https://doi.org/10.1016/S0145-2134(01)00232-0

Sternberg, K. J., Lamb, M. E., Esplin, P. W., & Baradaran, L. P. (1999). Using a scripted protocol in investigative interviews: A pilot study. *Applied Developmental Science, 3*(2), 70-76. https://doi.org/10.1207/s1532480xads0302_1

Sternberg, K. J., Lamb, M. E., Hershkowitz, I., Yudilevitch, L., Orbach, Y., Esplin, P. W., & Hovav, M. (1997). Effects of introductory style on children’s abilities to describe experiences of sexual abuse. *Child Abuse & Neglect, 21*(11), 1133-1146. https://doi.org/10.1016/S0145-2134(97)00071-9

Sternberg, K. J., Lamb, M. E., Orbach, Y., Esplin, P. W., & Mitchell, S. (2001). Use of a structured investigative protocol enhances young children's responses to free-recall prompts in the course of forensic interviews. *Journal of Applied Psychology, 86*(5), 997-1005. https://doi.org/10.1037/0021-9010.86.5.997

Sumampouw, N. E. J., Otgaar, H., La Rooy, D., & de Ruiter, C. (2019). The quality of forensic child interviewing in child sexual abuse cases in Indonesia. *Journal of Police and Criminal Psychology, 35*(2), 170-181. https://doi.org/10.1007/s11896-019-09342-5

Teoh, Y., & Lamb, M. E. (2010). Preparing children for investigative interviews: Rapport-building, instruction, and evaluation. *Applied Developmental Science, 14*(3), 154-163. https://doi.org/10.1080/10888691.2010.494463

Teoh, Y. S., & Lamb, M. E. (2013). Interviewer demeanor in forensic interviews of children. *Psychology, Crime & Law, 19*(2), 145-159. https://doi.org/10.1080/1068316X.2011.614610

Teoh, Y.-S., Pipe, M.-E., Johnson, Z. H., & Lamb, M. E. (2014). Eliciting accounts of alleged child sexual abuse: How do children report touch? *Journal of Child Sexual Abuse, 23*(7), 792-803. https://doi.org/10.1080/10538712.2014.950400

Teoh, Y., Yang, P., Lamb, M. E., & Larsson, A. S. (2010). Do human figure diagrams help alleged victims of sexual abuse provide elaborate and clear accounts of physical contact with alleged perpetrators? *Applied Cognitive Psychology, 24*(2), 287-300. https://doi.org/10.1002/acp.1564

Thierry, K. L., Lamb, M. E., & Orbach, Y. (2003). Awareness of the origin of knowledge predicts child witnesses’ recall of alleged sexual and physical abuse. *Applied Cognitive Psychology, 17*(8), 953-967. https://doi.org/10.1002/acp.933

Thierry, K. L., Lamb, M. E., Orbach, Y., & Pipe, M.-E. (2005). Developmental differences in the function and use of anatomical dolls during interviews with alleged sexual abuse victims. *Journal of Consulting and Clinical Psychology, 73*(6), 1125-1134. https://doi.org/10.1037/0022-006X.73.6.1125

Thoresen, C., Lønnum, K., Melinder, A., & Magnussen, S. (2009). Forensic interviews with children in CSA cases: A large-sample study of Norwegian police interviews. *Applied Cognitive Psychology, 23*(7), 999-1011. https://doi.org/10.1002/acp.1534

VanMeter, F., Henderson, H., Konovalov, H., Karni-Visel, Y., & Blasbalg, U. (2021). Children's narrative coherence in 'achieving best evidence' forensic interviews and courtroom testimony. *Psychology, Crime & Law, 29*(2), 203-221*.* https://doi.org/10.1080/1068316X.2021.2018438

Waterhouse, G. F., Ridley, A. M., Bull, R., La Rooy, D., & Wilcock, R. (2016). Dynamics of repeated interviews with children. *Applied Cognitive Psychology, 30*(5), 713-721. https://doi.org/10.1002/acp.3246

Waterhouse, G. F., Ridley, A. M., Bull, R., La Rooy, D., & Wilcock, R. (2018). Mapping repeated interviews. *Journal of Police and Criminal Psychology, 34*(4), 392-409. https://doi.org/10.1007/s11896-018-9288-7

Welbourne, P. (2002). Videotaped evidence of children: Application and implications of the memorandum of good practice. *British Journal of Social Work, 32*(5), 553-571. https://doi.org/10.1093/bjsw/32.5.553

Westcott, H. L., Kynan, S., & Few, C. (2006). Improving the quality of investigative interviews for suspected child abuse: A case study. *Psychology, Crime & Law, 12*(1), 77-96. https://doi.org/10.1080/10683160500036988

White, S., Strom, G. A., Santilli, G., & Halpin, B. M. (1986). Interviewing young sexual abuse victims with anatomically correct dolls. *Child Abuse & Neglect, 10*(4), 519-529. https://doi.org/10.1016/0145-2134(86)90057-8

Wolfman, M., Brown, D., & Jose, P. (2016). Taking stock: Evaluating the conduct of forensic interviews with children in New Zealand. *Psychology, Crime & Law, 22*(6), 581-598. https://doi.org/10.1080/1068316X.2016.1168426

Yi, M., Jo, E., & Lamb, M. E. (2016). Effects of the NICHD protocol training on child investigative interview quality in Korean police officers. *Journal of Police and Criminal Psychology, 31*(2), 155-163. https://doi.org/10.1007/s11896-015-9170-9

Yi, M., Jo. E., & Lamb, M. E. (2017). Assessing the effectiveness of NICHD protocol training focused on episodic memory training and rapport-building: A study of Korean police officers. *Journal of Police and Criminal Psychology, 32*(4), 279-288. https://doi.org/10.1007/s11896-016-9220-y

Yi, M., Lamb, M. E., & Jo, E. (2015). The quality of Korean police officers' investigative interviews with alleged sexual abuse victims as revealed by self-report and observation. *Journal of Police and Criminal Psychology, 30*(4), 274-281. https://doi.org/10.1007/s11896-014-9157-y
