# Supplementary material for: Forensic Interview Techniques in Child Sexual Abuse Cases: A Scoping Review
Source: Trauma Violence Abuse. 2023 Jun 5;25(2):1382–96. doi: 10.1177/15248380231177317 (PMC10913353; doi:10.1177/15248380231177317)
Supplement: sj-docx-2-tva-10.1177_15248380231177317 – Supplemental material for Forensic Interview Techniques in Child Sexual Abuse Cases: A Scoping Review [file sj-docx-2-tva-10.1177_15248380231177317.docx]

**Supplemental Appendix B**

**Description of characteristics of included studies**

| Study | Country | Type of victimization | Sample characteristics | Interviewer profession | Interview description |
| --- | --- | --- | --- | --- | --- |
| Ahern et al. (2015) | UK | CSA | *N* = 95; 4- to 13-year-old | Police officers | NICHD protocol vs MoGP |
| Ahern et al. (2014) | Israel | CSA or PA | *N* = 199; 3- to 13-year-old | Youth investigators | NICHD protocol vs Revised NICHD protocol |
| Ahern and Lamb (2017) | UK | CSA | *N* = 95; 4- to 13-year-old | Police officers | NICHD protocol vs MoGP |
| Ahern et al. (2018) | UK | CSA | *N* = 95; 4- to 13-year-old | Police officers | NICHD protocol vs MoGP |
| Åker and Johnson (2020) | Norway | CSA or PA | *N* = 82; 5- to 50-year-old with ID or ASD | Police officers | SI model |
| Aldridge and Cameron (1999) | UK | CSA | *N* = 27; 3- to 9-year-old | Jointly a police officer and a social worker | The interview uses a stepwise procedure through explicit and progressive broad stages |
| Aldridge et al. (2004) | UK | CSA | *N* = 90; 4- to 13-year-old | Police officers | NICHD protocol and HFD with structured questions |
| Alonzo-Proulx and Cyr (2016) | Canada | CSA | *N* = 116; 4- to 14-year-old | Police officers | Pre vs post-training in NICHD protocol |
| Alsaif et al. (2018) | Saudi Arabia | CSA | *N* = 2; 5-year-old, and 9-year-old | Forensic interviewers | NICHD protocol vs the general rules of forensic interviewing in a stepwise manner were ensured |
| Alves et al. (2019) | Brazil | CSA | *N* = 31; 3.1- to 17.1-year-old | Social workers and psychologists | No specific guidelines or protocols were followed |
| Anderson et al. (2014) | USA | CSA | *N* = 115; 3.1- to 18.3-year-old with ND, ADHD, DD, anxiety, or depression | Forensic interviewers | CornerHouse RATAC protocol with vs without narrative event practice rapport techniques |
| Andrews and Lamb (2014) | USA | CSA | *N* = 115; 3.8- to 12.5-year-old | Forensic interviewers | No specific guidelines or protocols were followed |
| Azzopardi et al. (2014) | Canada | CSA, PA, and/or neglect | *N* = 47; 3- to 12-year-old | Social workers and psychologists | NICHD protocol with modifications adapting to the developmental and emotional needs of the child |
| Baugerud et al. (2020) | Norway | CSA or PA | *N* = 207; 3- to 7-year-old | Police officers | SI model |
| Blasbalg et al. (2021) | Israel | CSA or PA | *N* = 202; 3- to 14-year-old | Youth investigators | Revised NICHD protocol |
| Bracewell (2018) | USA | CSA | *N* = 309; Age NR | Forensic interviewers | CACs of Texas protocol |
| Brubacher et al. (2013) | UK | CSA | *N* = 97; 5- to 13-year-old | Police officers | NICHD protocol vs MoGP |
| Burrows et al. (2017) | Australia | CSA | *N* = 161; 4- to 17-year-old | Police officers or CPS workers | SIM framework |
| Cantlon et al. (1996) | USA | CSA | *N* = 1535; 2- to 17-year-old and 5.5% with D | Nurses | “Allegation-blind” vs “allegation-informed” interview technique |

*(continued)*

| Study | Country | Type of victimization | Sample characteristics | Interviewer profession | Interview description |
| --- | --- | --- | --- | --- | --- |
| Castelli and Goodman (2014) | USA | CSA | *N* = 98; 4- to 17-year-old | Social workers | Semi-structured interview according to funnel approach |
| Cederborg et al. (2011) | Sweden | CSA or PA | *N* = 32; 5.4- to 23.7-year-old with DD, ID, ASD, or ID combined with ASD | Police officers | No specific guidelines or protocols were followed |
| Cederborg et al. (2008) | Sweden | CSA and/or PA | *N* = 19; 6.1- to 22-year-old DD, ADHD, DD combined with ASD or ASD | NR | No specific guidelines or protocols were followed |
| Cederborg and Lamb (2008) | Sweden | CSA or PA | *N* = 11; 6.1- to 22-year-old DD, ADHD or mild DD combined with ASD | Police officers | No specific guidelines or protocols were followed |
| Cederborg et al. (2000) | Sweden | CSA | *N* = 72; 4.2- to 12-year-old | Police officers | No specific guidelines or protocols were followed |
| Cheung (1997) | Hong Kong | CSA | NA | Social workers, police officers, and clinical psychologists | CSAIP |
| Cheung (2008) | USA | CSA | *N* = 90; 2- to 16-year-old | NR | CSAIP |
| Cheung and Boutté-Queen (2010) | USA | CSA | NA | CACs professionals | CSAIP with SACD and/or HFD |
| Cyr et al. (2012) | Canada | CSA | *N* = 163; 3- to 14-year-old | Police officers | Pre vs post-training in NICHD protocol |
| Cyr and Lamb (2009) | Canada | CSA | *N* = 166; 3- to 13-year-old | Police officers and social workers | Pre vs post-training in NICHD protocol |
| Davies et al. (2000) | UK | CSA | *N* = 36; 4- to 14-year-old | Police officers | MoGP |
| Dion and Cyr (2008) | Canada | CSA | *N* = 34; 6- to 14-year-old | Police officers and social workers | Pre vs post-training in NICHD protocol |
| Duron (2018a) | USA | CSA | *N* = 100; *M* = 11.20-year-old (*SD* = 3.9) | CACs professionals | CSAIP |
| Duron (2018b) | USA | CSA | *N* = 100; *M* = 11.94-year-old (*SD* = 3.81) | CACs professionals | CACs of Texas protocol |
| Earhart et al. (2014) | UK | CSA | *N* = 76; 4- to 13-year-old | Police officers and social workers | MoGP |
| Feltis et al. (2010) | Australia | CSA (82%) and NR (18%) | *N* = 34; 5- to 15-year-old | Police officers | The interview uses a stepwise procedure through explicit and progressive broad stages |
| Gagnon and Cyr (2017) | Canada | CSA | *N* = 55; 3- to 5-year-old | Police officers | NICHD protocol |
| Garcia et al. (2022) | Australia | CSA (90%), PA, or other | *N* = 328; 4- to 16-year-old | Police officers | SIM framework |
| Gudjonsson et al. (2010) | USA | CSA | *N* = 285; 3.5- to 17-year-old | Psychologist, social worker, and criminologist | Child interview protocol guide of the Children’s House |

*(continued)*

| Study | Country | Type of victimization | Sample characteristics | Interviewer profession | Interview description |
| --- | --- | --- | --- | --- | --- |
| Hamilton et al. (2016a) | Australia | CSA | *N* = 70; 5- to 16-year-old with Aboriginal children | Police officers | SIM framework |
| Hamilton et al. (2016b) | Australia | CSA | *N* = 70; 5- to 16-year-old with Aboriginal children | Police officers | SIM framework |
| Henderson and Lyon (2020) | USA | Predominantly CSA | *N* = 446; 2- to 18-year-old | CACs professionals | 10-Step protocol |
| Hershkowitz (2001) | Israel | CSA | *N* = 50; 4- to 13-year-old | Youth investigators | NICHD protocol |
| Hershkowitz (2002) | Israel | CSA | *N* = 50; 4- to 13-year-old | Youth investigators | NICHD protocol |
| Hershkowitz (2006) | Israel | CSA and/or PA | *N* = 26098; 2- to 14-year-old | Youth investigators | NICHD protocol |
| Hershkowitz (2009) | Israel | CSA | *N* = 71; 4- to 9-year-old | Youth investigators | NICHD protocol |
| Hershkowitz et al. (2017) | Israel | CSA or PA | *N* = 321; 3.29- to 14-year-old | Youth investigators | Revised NICHD protocol |
| Hershkowitz, Fisher et al. (2007) | Israel | CSA | *N* = 24; Age NR | Youth investigators | Pre vs post-training in NICHD protocol |
| Hershkowitz et al. (2005) | Israel | CSA or PA | *N* = 26408; 3- to 14-year-old | Youth investigators | NICHD protocol |
| Hershkowitz and Lamb (2020) | Israel | CSA or PA | *N* = 14874; 4- to 14-year-old | Youth investigators | NICHD protocol vs Revised NICHD protocol |
| Hershkowitz et al. (2021) | Israel | CSA or PA | *N* = 104; 3.5- to 13.63-year-old | Youth investigators | Revised NICHD protocol |
| Hershkowitz, Lamb, and Horowitz (2007) | Israel | CSA and/or PA | *N* = 40430; 3- to 14-year-old with D | Youth investigators | NICHD protocol |
| Hershkowitz et al. (2014) | Israel | CSA or PA | *N* = 426; 4- to 13-year-old | Youth investigators | NICHD protocol vs Revised NICHD protocol |
| Hershkowitz et al. (2013) | Israel | CSA or PA | *N* = 199; 4- to 13-year-old | Youth investigators | NICHD protocol vs Revised NICHD protocol |
| Hershkowitz et al. (2012) | Israel | CSA or PA | *N* = 299; 3- to 6-year-old | Youth investigators | NICHD protocol |
| Hershkowitz et al. (1997) | Israel | CSA | *N* = 20; 4- to 13-year-old | Forensic psychologists | No specific guidelines or protocols were followed |
| Hershkowitz, Lanes, and Lamb (2007) | Israel | CSA | *N* = 30; 7- to 12-year-old | Youth investigators | NICHD protocol |
| Hershkowitz et al. (2001) | Israel | CSA | *N* = 96; 4.4- to 13.4-year-old | Youth investigators | NICHD protocol with vs without MCR techniques |
| Hershkowitz et al. (2002) | Israel | CSA | *N* = 142; 4- to 13.5-year-old | Youth investigators | NICHD protocol with MCR vs PCR vs non-context reinstatement instructions |
| Hershkowitz et al. (2006) | Israel | CSA or PA | *N* = 100; 4- to 13-year-old | Youth investigators | NICHD protocol |
| Hershkowitz et al. (1998) | Israel | CSA | *N* = 51; 4.4- to 13.4-year-old | Youth investigators | Structured interview protocol an interview at the office and a follow-up interview at the scene of the alleged crime |

*(continued)*

| Study | Country | Type of victimization | Sample characteristics | Interviewer profession | Interview description |
| --- | --- | --- | --- | --- | --- |
| Hershkowitz and Terner (2007) | Israel | CSA | *N* = 40; 6- to 13-year-old | Youth investigators | NICHD protocol |
| Hlavka (2014) | USA | CSA | *N* = 100; 3- to 17-year-old | Forensic interviewer | APSAC protocol |
| Hlavka et al. (2010) | USA | CSA | *N* = 500; 2- to 17-year-old with NR D (82%), DD (5%), mental health diagnosis (2%), or unspecific D (11%) | Forensic interviewer | CornerHouse RATAC protocol with vs without SACD |
| Johnson et al. (2015) | Norway | CSA | *N* = 224; 3- to 16-year-old | Police officers, psychologist, and social workers | No specific guidelines or protocols were followed |
| Karni-Visel et al. (2021) | Israel | CSA and/or PA | *N* = 100; 3.46- to 13.90-year-old | Youth investigators | Revised NICHD protocol |
| Kask (2012) | Estonia | CSA and/or PA | *N* = 66; 4- to 14-year-old | Police officers | No specific guidelines or protocols were followed |
| Katz (2013) | Israel | Internet-related CSA | *N* = 20; 11- to 14-year-old | Youth investigators | NICHD protocol |
| Katz (2014) | Israel | CSA or PA | *N* = 12; 5- to 13-year-old | Youth investigators | NICHD protocol |
| Katz (2015) | Israel | CSA | *N* = 3; 5-year-old, 10-year-old, and 12-year-old | Youth investigators | Revised NICHD protocol |
| Katz and Barnetz (2014) | Israel | CSA or PA | *N* = 224; 5- to 13-year-old | Youth investigators | NICHD protocol |
| Katz and Barnetz (2018) | Israel | CSA or PA | *N* = 224; 4- to 14-year-old | Youth investigators | NICHD protocol |
| Katz et al. (2014) | Israel | CSA | *N* = 125; 4- to 14-year-old | Youth investigators | NICHD protocol with vs without the alleged crime drawing |
| Katz and Hershkowitz (2010) | Israel | CSA | *N* = 125; 4- to 14-year-old | Youth investigators | NICHD protocol with vs without the alleged crime drawing |
| Katz and Hershkowitz (2012) | Israel | CSA | *N* = 71; 4- to 9-year-old | Youth investigators | NICHD protocol |
| Katz and Hershkowitz (2013) | Israel | CSA | *N* = 56; 5- to 15-year-old | Youth investigators | NICHD protocol |
| Katz et al. (2021) | Israel | CSA | *N* = 60; 10- to 14-year-old | Youth investigators | NICHD protocol |
| Katz et al. (2020) | Israel | CSA | *N* = 30; 7- to 14-year-old | Youth investigators | NICHD protocol |
| Katz et al. (2012) | Israel | CSA or PA | *N* = 40; 3.1- to 13.5-year-old | Youth investigators | NICHD protocol |
| Katz et al. (2016) | Israel | CSA | *N* = 97; 3- to 14-year-old | Youth investigators | NICHD protocol |
| Kim et al. (2020) | South Korea | SA | *N* = 86; 7- to 49-year-old | Police officers | NICHD protocol |

*(continued)*

| Study | Country | Type of victimization | Sample characteristics | Interviewer profession | Interview description |
| --- | --- | --- | --- | --- | --- |
| Korkman, Santtila, Drzewiecki, and Sandnabba (2008) | Finland | CSA | *N* = 43; 3- to 8-year-old | Clinical mental health professionals | No specific guidelines or protocols were followed |
| Korkman et al. (2006) | Finland | CSA | *N* = 12; *M* = 5.83-year-old (*SD* = 1.54) | Police officers, psychiatrists, psychologists | No specific guidelines or protocols were followed |
| Korkman, Santtila, Westeraker, and Sandnabba (2008) | Finland | CSA | *N* = 43; 3- to 8-year-old | Clinical mental health professionals | No specific guidelines or protocols were followed |
| Lafontaine and Cyr (2016a) | Canada | CSA | NA | Police officers | NICHD protocol |
| Lafontaine and Cyr (2016b) | Canada | CSA | *N* = 114; 3- to 17-year-old | Police officers | NICHD protocol |
| Lamb and Fauchier (2001) | USA | CSA | *N* = 7; 5.5- to 8.9-year-old | Forensic interviewers | No specific guidelines or protocols were followed |
| Lamb and Garretson (2003) | Israel, UK, and USA | CSA and/or PA | *N* = 672; 4- to 14-year-old | Forensic interviewers | NICHD protocol vs no specific guidelines or protocols were followed |
| Lamb et al. (1996) | USA | CSA | *N* = 24; 4- to 12-year-old | CPS workers | With vs without SACD |
| Lamb et al. (2007) | Israel | CSA | *N* = 43; 3.5- to 13.67-year-old | Youth investigators | NICHD protocol |
| Lamb et al. (2009) | UK | CSA | *N* = 100; 4- to 13-year-old | Police officers | NICHD protocol vs MoGP |
| Lamb, Sternberg, and Esplin (2000) | USA | CSA | *N* = 145; 4- to 12-year-old | Social workers | No specific guidelines or protocols were followed |
| Lamb et al. (2002) | USA | CSA and/or PA | *N* = 74; 4- to 12-year-old | Police officers | NICHD protocol |
| Lamb, Sternberg, Orbach, Esplin et al. (2003) | UK and USA | CSA | *N* = 130; 4- to 8-year-old | Police officers | NICHD protocol |
| Lamb, Sternberg, Orbach, Hershkowitz, and Horowitz (2003) | Israel | CSA victims or witnesses | *N* = 52; 5- to 14-year-old | Youth investigators | NICHD protocol |
| Lamb, Sternberg, Orbach et al. (2000) | Israel | CSA | *N* = 192; 4- to 14.25-year-old | Youth investigators | Pre vs post-training in NICHD protocol |
| Leach et al. (2017) | Australia | CSA | *N* = 527; 3- to 16-year-old | CPS workers and/or police officers | SIM framework |
| Leander (2010) | Sweden | CSA | *N* = 27; 5- to 17-year-old | Police officers | No specific guidelines or protocols were followed |
| Lee and Kim (2020) | South Korea | CSA | *N* = 137; 41.6% < 13-year-old and 40.1% with D | Police officers | NICHD protocol |
| Lewy et al. (2015) | Canada | CSA | *N* = 90; 4- to 13-year-old | Police officers | Pre vs post-training in NICHD protocol |

*(continued)*

| Study | Country | Type of victimization | Sample characteristics | Interviewer profession | Interview description |
| --- | --- | --- | --- | --- | --- |
| Lindholm et al. (2014) | Sweden | VoT | *N* = 12; Adolescents | Police officers | No specific guidelines or protocols were followed |
| Lindholm et al. (2015) | Sweden | VoT | *N* = 24; 14- to 21-year-old | Police officers | No specific guidelines or protocols were followed |
| Lippert et al. (2009) | USA | CSA | *N* = 987; *M* = 9.91-year-old (*SD* = 4.06) | Forensic interviewers | APSAC protocol |
| Malloy et al. (2011) | UK and USA | CSA | *N* = 204; 5- to 13-year-old | Police officers | NICHD protocol |
| Malloy et al. (2013) | UK and USA | CSA | *N* = 204; 5- to 13-year-old | Police officers | NICHD protocol |
| Malloy et al. (2016) | USA | CSA | *N* = 49; 3- to 5-year-old | Police officers | NICHD protocol |
| Melkman et al. (2017) | Israel | CSA | *N* = 1563; 3- to 14-year-old with or without ID | Youth investigators | NICHD protocol |
| Myklebust and Bjørklund (2010) | Norway | CSA | *N* = 100; 6- to 16-year-old | Police officers | No specific guidelines or protocols were followed |
| Orbach, Hershkowitz, Lamb, Sternberg, Esplin, and Horowitz (2000) | Israel | CSA | *N* = 55; 4- to 13-year-old | Youth investigators | Pre vs post-training in NICHD protocol |
| Orbach, Hershkowitz, Lamb, Sternberg, and Horowitz (2000) | Israel | CSA | *N* = 96; 4- to 13.5-year-old | Youth investigators | Structured interview protocol in office vs structured interview protocol in office and a follow-up interview at the scene of the alleged crime |
| Orbach and Lamb (1999) | USA | CSA | *N* = 1; 13-year-old | Sheriff | No specific guidelines or protocols were followed |
| Orbach and Lamb (2000) | USA | CSA | *N* = 2; 5-year-old and 15-year-old | Police officers | NICHD protocol |
| Orbach and Lamb (2001) | USA | CSA | *N* = 1; 5-year-old | Psychologist | No specific guidelines or protocols were followed |
| Orbach and Lamb (2007) | UK and USA | CSA | *N* = 250; 4- to 10-year-old | Police officers | NICHD protocol |
| Patterson and Pipe (2009) | New Zealand | CSA and/or PA | *N* = 24; 3- to 6-year-old | Social worker | The general rules of forensic interviewing in a stepwise manner were ensured |
| Peixoto et al. (2017) | Portugal | CSA and/or PA | *N* = 137; 3- to 17-year-old | Judges | No specific guidelines or protocols were followed |
| Peixoto et al. (2016) | Portugal | CSA | *N* = 33; 4- to 6-year-old | NR | NICHD protocol |
| Phillips et al. (2012) | UK | CSA | *N* = 21; 5- to 15-year-old | Police officers | ABE protocol |
| Pipe et al. (2013) | USA | CSA | *N* = 760; 2.8- to 13.97-year-old | Police officers | Pre vs post-training in NICHD protocol |
| Price et al. (2016) | UK | CSA | *N* = 94; 4- to 13-year-old | Police officers | NICHD protocol vs MoGP |
| Price and Roberts (2011) | Canada | CSA, PA, and/or neglect | *N* = 117; 4- to 16-year-old | CPS workers or police officers | Pre vs post-training in NICHD protocol |

*(continued)*

| Study | Country | Type of victimization | Sample characteristics | Interviewer profession | Interview description |
| --- | --- | --- | --- | --- | --- |
| Richardson et al. (2019) | UK | SA | *N* = 18; 12- to 35-year-old | Police officers | ABE protocol |
| Santtila et al. (2004) | Finland | CSA | *N* = 12; *M* = 5.83-year-old (*SD* = 1.54) | Police officers, psychiatrists, psychologists | No specific guidelines or protocols were followed |
| Schaeffer et al. (2011) | USA | CSA | *N* = 191; 3- to 18-year-old | Forensic interviewers | CornerHouse RATAC protocol |
| Sim and Lamb (2013) | UK | CSA | *N* = 97; 4- to 13-year-old | Police officers | NICHD protocol vs MoGP |
| Smith et al. (2009) | Australia | CSA, PA, and/or neglect | NA | Police officers | SIM framework |
| Sternberg, Lamb, Davies, and Westcott (2001) | UK | CSA | *N* = 119; 4- to 13-year-old | Police officers | MoGP |
| Sternberg et al. (1999) | USA | CSA | *N* = 44; 4- to 12-year-old | Police officers | No specific guidelines or interview protocols were followed vs a scripted protocol for rapport building: direct or open-ended |
| Sternberg et al. (1997) | Israel | CSA | *N* = 51; 4.5- to 12.9-year-old | Youth investigators | Scripted protocol for rapport building: direct vs open-ended |
| Sternberg, Lamb, Orbach et al. (2001) | USA | CSA | *N* = 100; 4- to 12-year-old | Police officers | Pre vs post-training in NICHD protocol |
| Sumampouw et al. (2019) | Indonesia | CSA | *N* = 22; 4- to 14-year-old | Police officers | No specific guidelines or protocols were followed |
| Teoh and Lamb (2010) | Malaysia | CSA | *N* = 75; 5- to 15-year-old | Police officers | The general rules of forensic interviewing in a stepwise manner were ensured |
| Teoh and Lamb (2013) | Malaysia | CSA | *N* = 75; 5- to 15-year-old | Police officers | The general rules of forensic interviewing in a stepwise manner were ensured |
| Teoh et al. (2014) | USA | CSA | *N* = 192; 4- to 13-year-old | Police officers | NICHD protocol |
| Teoh et al. (2010) | UK | CSA | *N* = 88; 4- to 13-year-old | Police officers | NICHD protocol and HFD with structured questions |
| Thierry et al. (2003) | USA | CSA and/or PA | *N* = 116; 3- to 11-year-old | Police officers | NICHD protocol |
| Thierry et al. (2005) | USA | CSA | *N* = 178; 3- to 12-year-old | CPS workers or police officers | Forensic interview with SACD. The general rules of forensic interviewing in a stepwise manner were ensured |
| Thoresen et al. (2009) | Norway | CSA | *N* = 195; 3.4- to 14.2-year-old | Judges, police officers, psychiatrists, psychologists, and social worker | No specific guidelines or protocols were followed |
| VanMeter et al. (2021) | UK | CSA | *N* = 80; 7- to 15-year-old | Police officers | ABE protocol |
| Waterhouse et al. (2016) | UK | CSA and/or PA | *N* = 21; 3- to 14-year-old | Police officers and social workers | No specific guidelines or protocols were followed |
| Waterhouse et al. (2018) | UK | CSA or PA | *N* = 4; 5- to 9-year-old | Police officers and social workers | Guidance on Joint Investigative Interviewing of Child Witnesses |

*(continued)*

| Study | Country | Type of victimization | Sample characteristics | Interviewer profession | Interview description |
| --- | --- | --- | --- | --- | --- |
| Welbourne (2002) | UK | CSA | *N* = 36; 4- to 17-year-old | Police officers and social workers | MoGP |
| Westcott et al. (2006) | UK | CSA victims or witnesses | *N* = 51; 3.92% with D | Police officers and social workers | MoGP |
| White et al. (1986) | USA | CSA | *N* = 50; 2- to 5.6-year-old | Psychologists and social workers | SACD protocol |
| Wolfman et al. (2016) | New Zealand | CSA | *N* = 93; 6- to 16-year-old | Police officers and social workers | SCWI model |
| Yi et al. (2016) | South Korea | CSA | *N* = 36; 5- to 14-year-old | Police officers | Pre vs post-training in NICHD protocol |
| Yi et al. (2017) | South Korea | CSA | NA | Police officers | Pre vs post-training in NICHD protocol |
| Yi et al. (2015) | South Korea | CSA | *N* = 45; 3- to 12-year-old | Police officers | NICHD protocol |

*Notes.* ABE = Achieving Best Evidence; ADHD = Attention Deficit Hyperactivity Disorder; APSAC = American Professional Society on the Abuse of Children; ASD = Autism Spectrum Disorder; CACs = Children’s Advocacy Centers; CPS = Child Protective Service; CSA = Child Sexual Abuse; CSAIP = Child Sexual Abuse Interview Protocol; D = Disabilities; DD = Developmental Disorder; HFD = Human Figure Diagram; ID – Intellectual Disabilities; MCR = Mental Context Reinstatement; MoGP = Memorandum of Good Practice; NA = Not Applicable; ND – Non Disabilities; NICHD = National Institute for Child Health and Human Development; NR = Not Reported; PA = Physical Abuse; PCR = Physical Context Reinstatement; RATAC = Rapport, Anatomy Identification, Touch inquiry, Abuse scenario, and Closure; SA = Sexual Assault; SACD = Sexually Anatomical Correct Dolls; SCWI = Specialist Child Witness Interviewing; SI = Sequential Interview; SIM = Standard Interview Method; UK = United Kingdom; USA = United States of America; VoT = Victim of Trafficking for Sexual Exploitation
